# Efficiency of the four proteasome subtypes to degrade ubiquitinated or oxidized proteins

**DOI:** 10.1038/s41598-020-71550-5

**Published:** 2020-09-25

**Authors:** Joanna Abi Habib, Etienne De Plaen, Vincent Stroobant, Dusan Zivkovic, Marie-Pierre Bousquet, Benoît Guillaume, Khadija Wahni, Joris Messens, Antonia Busse, Nathalie Vigneron, Benoit J. Van den Eynde

**Affiliations:** 1 Ludwig Institute for Cancer Research, Brussels, 1200 Belgium; 2 de Duve Institute, UCLouvain, Brussels, 1200 Belgium; 3 WELBIO (Walloon Excellence in Life Sciences and Biotechnology), Brussels, 1200 Belgium; 4 Institut de Pharmacologie et Biologie Structurale, IPBS, Université de Toulouse, CNRS, UPS, Toulouse, France; 5 Centre hospitalier de Jolimont, service de biochimie médicale, La Louvière, Belgium; 6 Structural Biology Research Center, VIB, 1050 Brussels, Belgium; 7 Structural Biology Brussels Laboratory, Vrije Universiteit Brussel, 1050 Brussels, Belgium; 8 Brussels Center for Redox Biology, 1050 Brussels, Belgium; 9 Medizinische Klinik III, Hämatologie, Onkologie und Tumorimmunologie Charité – Universitätsmedizin Berlin, corporate member of Freie Universität Berlin, Humboldt-Universität Zu Berlin, and Berlin Institute of Health, Campus Benjamin Franklin, Hindenburgdamm 30, 12203 Berlin, Germany; 10 Partner Site Berlin and German Cancer Research Center (DKFZ), German Cancer Consortium (DKTK), Heidelberg, Germany

**Keywords:** Ubiquitylated proteins, Proteolysis, Protein folding, Proteasome, Homeostasis

## Abstract

The proteasome is responsible for selective degradation of proteins. It exists in mammalian cells under four main subtypes, which differ by the combination of their catalytic subunits: the standard proteasome (β1–β2–β5), the immunoproteasome (β1i–β2i–β5i) and the two intermediate proteasomes (β1–β2–β5i and β1i–β2–β5i). The efficiency of the four proteasome subtypes to degrade ubiquitinated or oxidized proteins remains unclear. Using cells expressing exclusively one proteasome subtype, we observed that ubiquitinated p21 and c-­myc were degraded at similar rates, indicating that the four 26S proteasomes degrade ubiquitinated proteins equally well. Under oxidative stress, we observed a partial dissociation of 26S into 20S proteasomes, which can degrade non-ubiquitinated oxidized proteins. Oxidized calmodulin and hemoglobin were best degraded in vitro by the three β5i-containing 20S proteasomes, while their native forms were not degraded. Circular dichroism analyses indicated that ubiquitin-independent recognition of oxidized proteins by 20S proteasomes was triggered by the disruption of their structure. Accordingly, β5i-containing 20S proteasomes degraded unoxidized naturally disordered protein tau, while 26S proteasomes did not. Our results suggest that the three β5i-containing 20S proteasomes, namely the immunoproteasome and the two intermediate proteasomes, might help cells to eliminate proteins containing disordered domains, including those induced by oxidative stress.

## Introduction

The turnover of intracellular proteins is essential to maintain protein homeostasis in cells. It can occur by autophagy through the delivery of proteins to lysosomes, or by proteolysis by the proteasome, a tightly regulated complex that selectively degrades undesirable or damaged proteins^1, 2^ and generates small peptides to be presented on major histocompatibility complex (MHC) class I molecules and recognized by cytolytic T lymphocytes in the process of immune surveillance^3^.

The proteasome is a large multi-catalytic protease complex. Its basal structure, called 20S proteasome, is composed of two copies of fourteen different subunits assembled into four hetero-heptameric rings that delimit a cylindrical complex inside which proteins are degraded. The two identical outer rings are composed of α1–α7 subunits and form the gate, which regulates the entry of intracellular proteins inside the catalytic chamber of the 20S proteasome. The two inner rings are each composed of seven β-subunits (β1–β7), three of which bear a catalytic N-terminal threonine that initiates peptide bond hydrolysis^4^. In the standard proteasome (SP), which is the most abundant proteasome subtype in most cell types at steady state, the three constitutively expressed catalytic subunits are β1 (PSMB6), β2 (PSMB7) and β5 (PSMB5). In immune cells and in cells exposed to inflammatory cytokines, these three catalytic subunits are replaced by their inducible counterparts β1i (PSMB9, LMP2), β2i (PSMB10, MECL1) and β5i (PSMB8, LMP7), forming a second subtype of proteasome called the immunoproteasome (IP)^5^. Two additional proteasome subtypes expressing a mix of constitutive and inducible subunits are also found in normal tissues and in some human cancer cell lines: the intermediate proteasome β5i (hereafter called SIP for single intermediate proteasome), which contains β1, β2, β5i, and the intermediate proteasome β1i–β5i (called DIP for double intermediate proteasome), which contains β1i and β5i along with β2^6^.

The different proteasome subtypes are found either as free 20S particles or associated with regulatory particles (RP) that interact with the N-terminal tails of the α-subunits to facilitate opening of the gate^7^. These RP include PA28αβ (or 11S RP), PA28γ, PA200 and the 19S RP (or PA700), which associates with 20S proteasomes to form 26S proteasomes, which operate the ATP- and ubiquitin-dependent degradation of intracellular proteins. Ubiquitinated substrates are targeted to 26S proteasomes through interactions between ubiquitin and the different ubiquitin receptors of the 19S regulator. This step is followed by conformational modifications that favor translocation of the protein substrate coupled with its unfolding, deubiquitination and the opening of the proteasome gate^8–11^.

The catalytic properties of each proteasome subtype are determined by its combination of catalytic subunits. Each catalytic subunit has a different cleavage specificity dictated by its structure and the nature of amino acids that line the catalytic pocket. Three major catalytic activities were described: the caspase-like, the trypsin-like and the chymotrypsin-like activities, which respectively cleave after acidic, basic and hydrophobic residues. The β1 subunit has caspase-like activity, whereas β1i rather cleaves after small hydrophobic or branched-chain amino acids, with the so-called branched amino­acid preferring (BrAAP) activity. Subunits β2i and β2 both display trypsin-like activity, β2i cleaving more efficiently than β2. Finally, the two homologous subunits β5 and β5i display chymotrypsin-like activity, β5i being more efficient than β5^6, 12, 13^. As a result, each proteasome subtype produces a unique repertoire of antigenic peptides^6, 14–16^. Because the IP has a higher propensity to cleave after hydrophobic and basic residues, it was predicted to produce peptides that bind more efficiently to MHC class I^15, 17, 18^. Aside from the production of MHC class I peptides, several other proposed functions of the IP, such as activation of the NF­κB pathway^19, 20^ or selective turnover of ubiquitinated proteins^21^, remain controversial^15, 22–24^.

Seifert et al*.* showed that the IP was more efficient than the SP at degrading ubiquitinated proteins and suggested a specific role of the IP to rapidly degrade newly synthesized defective proteins that accumulate following IFNγ exposure and are both ubiquinated and oxidized^21^. These results were soon challenged by Nathan et al*.* who showed in a very similar set of experiments that the SP and the IP did not differ in their capacity to degrade ubiquitinated proteins^23^. Because of these discrepancies, it is unclear whether the IP is more efficient than the SP in the ubiquitin-dependent degradation of proteins. Furthermore, the role of the intermediate proteasomes in this matter has never been explored. In order to address these issues, we first set up an experimental approach that is independent of the use of IFNγ, to study the degradation of ubiquitinated proteins by 26S SP, IP and intermediate proteasomes. We then studied how the four proteasome subtypes degrade oxidized proteins. Because in our hands the turnover of oxidized proteins could not be reliably assessed in cells, and because free 20S proteasomes were shown to degrade non-ubiquitinated oxidized proteins that accumulate upon oxidative stress^25–27^, we set up an in vitro approach to study the ATP- and ubiquitin-independent degradation of oxidized proteins by the four 20S proteasome subtypes. Finally, we explored the rules that dictate this unconventional degradation of oxidized proteins.

## Results

### Ubiquitinated proteins are degraded at similar rates by the four 26S proteasome subtypes

To examine the relative efficiency of the four proteasome subtypes to degrade ubiquitinated proteins, we used human HEK293-EBNA cell lines (hereafter called 293) expressing exclusively either the standard proteasome (SP), the immunoproteasome (IP), the intermediate proteasome β5i (SIP) or the intermediate proteasome β1i–β5i (DIP)^6^. These cell lines were previously obtained following successive transfection of a parental ­293 cell line, which exclusively expresses the SP (hereafter called 293 SP), with strong expression vectors encoding the different inducible subunits, generating the 293 SIP, DIP and IP. These three cell lines maintained similar levels of transcription of the constitutive subunits PSMB6 (β1), PSMB7 (β2) and PSMB5 (β5) as the parental 293 SP as shown by RNA-seq analysis (Fig. S1a). However, the stable overexpression of the inducible subunits PSMB8 (β5i), PSMB9 (β1i) and PSMB10 (β2i) (Fig. S1a,b), together with their preferential incorporation into nascent proteasomes^28–30^, ensured a complete replacement of SP by SIP in β5i-transfected cells, by DIP in β1i–β5i-transfected cells, or by IP in β1i–β2i–β5i-transfected cells. Indeed, LC–MS/MS analysis of proteasomes purified from these cell lines using a sucrose gradient based technique adapted from Schmidtke et al.^31^, which is not specific for a given proteasome subtype, showed that 293 SP, SIP, DIP and IP cell lines contained exclusively SP, SIP, DIP and IP respectively (Fig. S5b). The lack of incorporation of constitutive subunits in the proteasomes of 293 SIP, DIP and IP cell lines appears to trigger their degradation, as the western blot analysis of the total lysates did not show any trace of unincoroporated subunits (Fig. S1b). Because these cell lines have the same background and only differ in their composition of proteasome catalytic subunits, they are ideally suited to compare the intracellular degradation of ubiquitinated proteins by 26S SP, IP and intermediate proteasomes.

**Figure 1:**
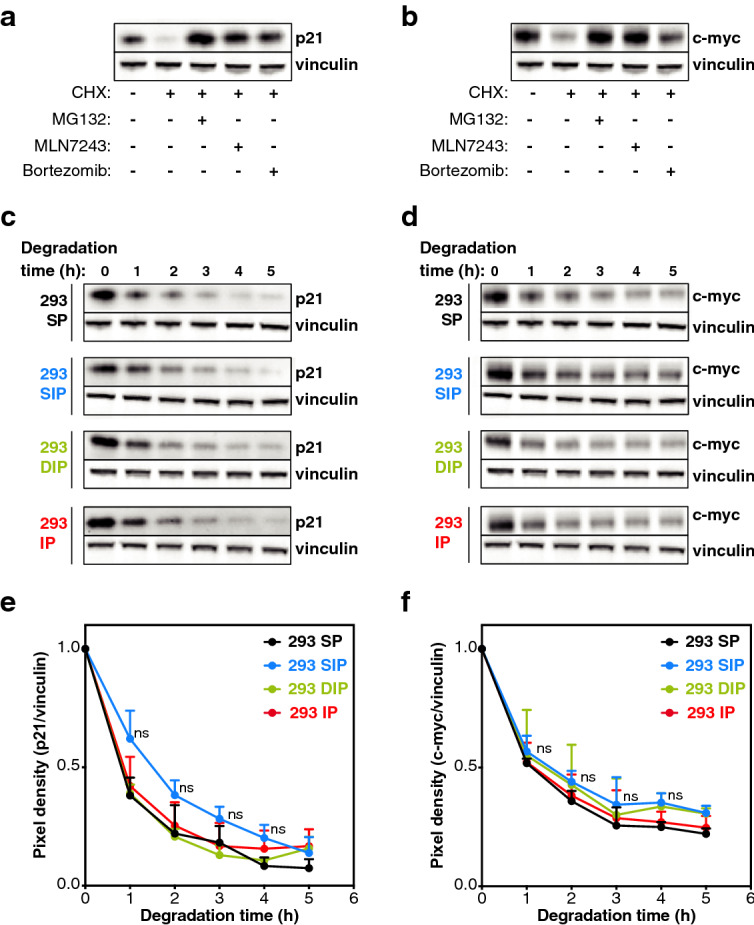
Degradation of ubiquitinated proteins by the standard proteasome (SP), the intermediate proteasome β5i (SIP), the intermediate proteasome β1i–β5i (DIP) and the immunoproteasome (IP). (**a**) p21 and (**b**) c-myc are degraded in a proteasome and ubiquitin-dependent manner. Western blot analysis of lysates of 293 SP cells treated or not with cycloheximide (CHX) alone or in combination with MLN7243, an inhibitor of the ubiquitin-activating enzyme, or with MG132 or bortezomib, two proteasome inhibitors. Cells were treated with MLN7243, MG132 or Bortezomib 30 min prior to the 5 h treatment with cycloheximide. (**c**, **d**) Western blot analysis of the kinetics of degradation of (**c**) p21 and (**d**) c-myc in 293 cell lines expressing the different proteasome subtypes. (**e**, **f**) Densitometric evaluation of the kinetics of the degradation of (**e**) p21 and (**f**) c-myc in the four different cell lines. All values (+ SD) are collected from three independent experiments. Full-length images for (a-d) are presented in Fig. S10.

As a first step, we monitored the half-life of the pool of ubiquitinated proteins in the four 293 cells treated with MLN7243, an inhibitor of the ubiquitin-activating enzyme^32^. The decay of ubiquitinated proteins was similar in the four cell lines, and was prevented to a similar extent by the proteasome inhibitor MG132, suggesting equal degradation of ubiquitinated proteins by the four proteasome subtypes (Fig. S2). Of note, MG132 did not fully prevent the decay of ubiquitinated proteins, which also resulted from the action of deubiquitinases, whose inhibition by PR619 increased the amount of ubiquitinated proteins (Fig. S2a). We then studied the degradation of two specific protein substrates: the cell cycle regulator p21 (CIP1/WAF1) and the transcription factor c-­myc. We first verified that the degradation of these two proteins was proteasome and ubiquitin-dependent in our 293 cells: it was efficiently blocked by proteasome inhibitors MG132 and bortezomib, and by ubiquitin-activating enzyme inhibitor MLN7243 (Fig. 1a,b). Ubiquitination of these two proteins was confirmed by isolating ubiquitinated proteins using the tandem ubiquitin binding entity (TUBE)-pull down assay (Fig. S3a)^33^ and detecting p21 or c­-myc by western blotting: ubiquitinated p21 and c-myc were detected and they were stabilized after treatment with proteasome inhibitor MG132 (Fig. S3b,c). Finally, we also checked that the four 293 cell lines did not differ in their expression of E1 ubiquitin-activating enzymes, E2 ubiquitin-conjugating enzymes and E3 ubiquitin ligases. RNA-seq data showed similar levels of transcripts for the E1 and E2 enzymes that are associated with the ubiquitin proteasome system (Fig. S4). Likewise, the transcripts associated with the E3 ubiquitin ligases responsible for ubiquitination of p21 and c­-myc^34–39^ were similarly expressed in the four 293 cell lines (Fig. S4). These cell lines therefore appear as a reliable model system to determine whether the four 26S proteasome subtypes differ in their ability to degrade ubiquitinated proteins, using p21 and c­-myc as paradigms.

We treated the four 293 cell lines expressing SP, SIP, DIP or IP with cycloheximide, and monitored p21 and c-myc degradation by western blot. We observed a similar rate of degradation of ubiquitinated p21 and c-myc in the four cell lines (Fig. 1c–f). Thus, SP and IP are equally efficient at degrading ubiquitinated proteins, confirming the findings of Nathan et al*.*^23^. Furthermore, the two intermediate proteasomes degrade ubiquitinated proteins at a rate similar to SP and IP.

### Immuno and intermediate 20S proteasomes degrade oxidized proteins more efficiently than standard 20S proteasomes

We tried to use the four 293 lines to study the degradation of oxidized proteins*.* However, the intracellular assays available to quantify oxidized proteins in cells, such as the oxyblots, proved unreliable in our hands.

**Figure 2:**
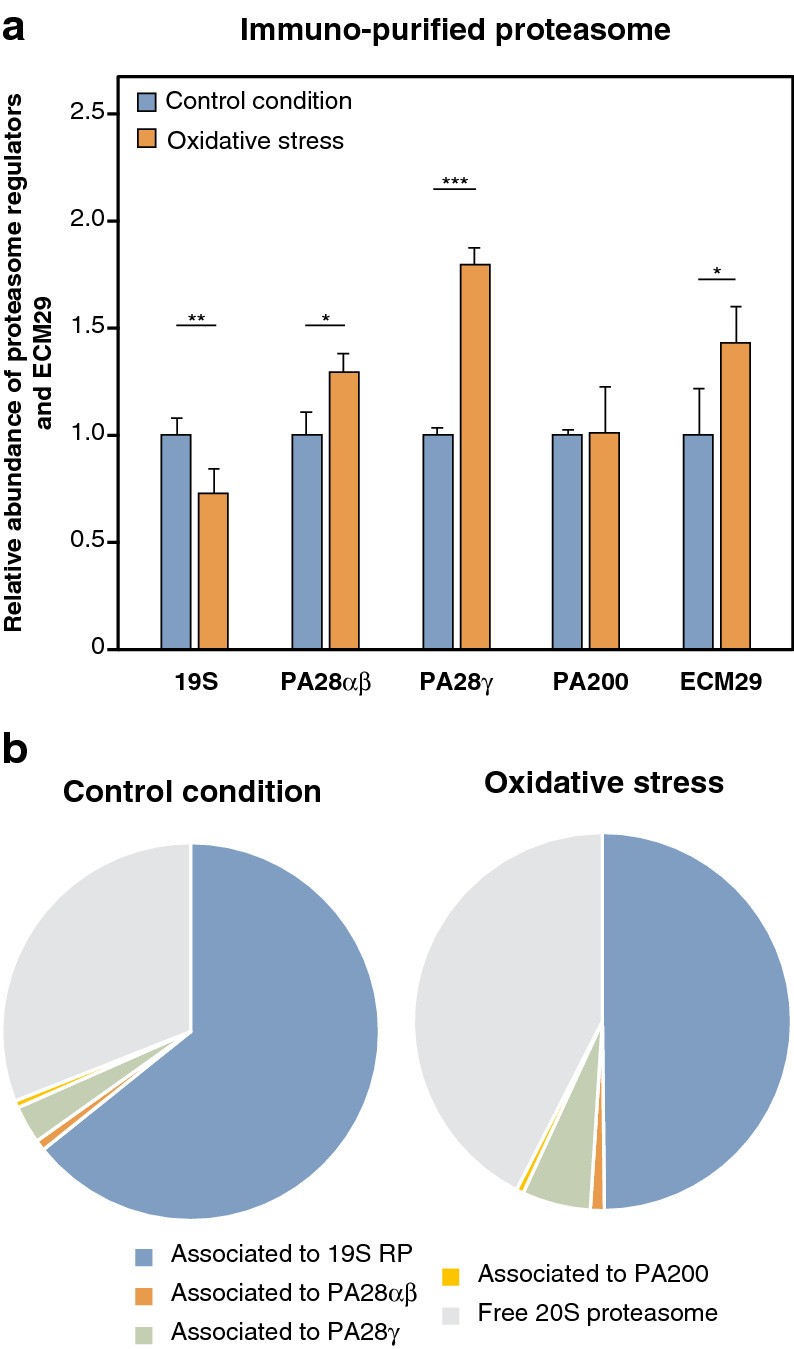
Oxidative stress dissociates 26S proteasomes into free 20S proteasomes. (**a**) Relative abundance of the different proteasome regulators and ECM29 bound to 20S proteasomes under oxidative stress induced by H_2_O_2_. 293 SP cells were treated with or without 2 mM of H_2_O_2_ for 30 min, proteins were cross-linked in vivo with formaldehyde, proteasomes were immuno-purified using the anti-α2 antibody (MCP21) and the different proteasome regulators were analyzed using label-free quantitative MS along with ECM29. The abundance of 20S interactors were measured in control and oxidized conditions, normalized to the abundance of 20S, and then the oxidized condition was normalized to the control condition. All values are means of three independent experiments + SD. (**b**) Pie chart showing the proportion of free 20S proteasome and of the different regulators associated to 20S proteasomes under control conditions (left panel) and oxidative stress (right panel).

During oxidative stress, proteasome-mediated degradation of oxidized proteins possibly occurs independently from the 19S RP and the ubiquitination system^25–27^. Moreover, previous work indicated that, under oxidative stress, 26S proteasomes are unstable and dissociate into 20S proteasomes^40–42^. Using quantification by label-free nano LC–MS/MS of proteasome-interacting proteins^43–45^, we also observed an increased proportion of 20S proteasomes with an increased binding to ECM29, a proteasome-interacting particle suggested to promote 26S proteasome dissociation (Fig. 2)^41, 42^. Accordingly, we observed a concomitant decrease in 26S proteasome (Fig. 2). These results confirm that 26S proteasomes are unstable upon oxidative stress and dissociate into free 20S proteasomes. This, together with the notion that 20S proteasomes can degrade oxidized proteins in an ubiquitin-independent manner^25, 27^ prompted us to use purified 20S proteasomes for in vitro digestion of oxidized proteins.

**Figure 3:**
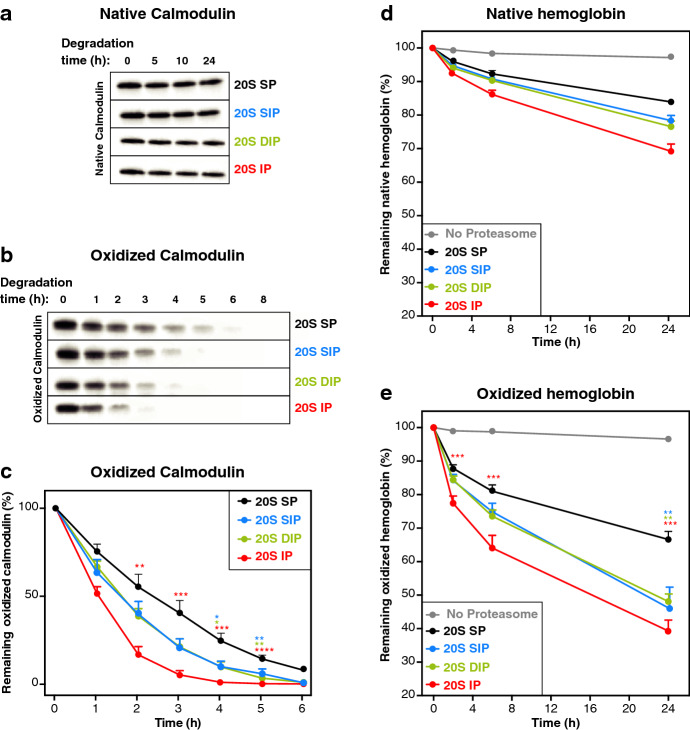
More efficient degradation of oxidized proteins by the 20S immunoproteasome (IP) and the two 20S intermediate proteasomes β5i (SIP) and β1i­-β5i (DIP). (**a**–**c**) Western blot analysis of the kinetics of degradation of (**a**) native and (**b**) oxidized calmodulin by the four 20S proteasome subtypes. (**c**) Densitometric evaluation of the kinetics of the degradation of the oxidized calmodulin by the four proteasome subtypes. All values (+ SEM) are collected from seven independent experiments using three different batches of purified proteasomes. (**d**, **e**) Kinetics of degradation of tritium-labeled (**d**) native and (**e**) oxidized hemoglobin. These two forms of hemoglobin were incubated with the four 20S proteasome subtypes. To monitor protein degradation, samples were collected at different time points, precipitated with trichloroacetic acid (TCA) and the radioactivity present in the supernatant was measured. Remaining hemoglobin was quantified by subtracting the radioactivity measured in the supernatant from the total radioactivity. Proteins were oxidized by 24 h incubation in 50 mM H_2_O_2_. All values are means (+ SEM) of seven independent experiments using three different batches of purified proteasomes. Full-length images for (a-b) are presented in Fig. S11.

20S proteasomes were purified from the four 293 lines using a sucrose gradient based technique adapted from Schmidtke et al.^31^. We used LC–MS/MS and silver staining of polyacrylamide gels to evaluate the purity of our preparations and confirm that they contained almost exclusively 20S proteasome subunits (Fig. S5a). LC­-MS/MS also showed that each proteasome preparation solely comprises the expected proteasome subtype (Fig. S5b). Using the small peptide GTPEGLYL, which is derived from the RPT2 subunit of the 19S RP and was shown to open the proteasome gate, we observed that the gate of our 20S proteasomes displayed a closed conformation (Fig. S5c)^46^. Finally, we used fluorogenic substrates to confirm that each proteasome preparation displayed the expected activities (Fig. S5d)^6, 13^. We then incubated native and H_2_O_2_-oxidized calmodulin with these 20S proteasomes and evaluated the degradation of calmodulin on immunoblots. As expected, native calmodulin was not degraded (Fig. 3a). Indeed, full-length, folded proteins are generally targeted to 26S proteasomes in an ubiquitin-dependent manner and are therefore resistant to degradation by 20S proteasomes. However, H_2_O_2_­-oxidized calmodulin was degraded by all four 20S proteasome subtypes, with intermediate proteasomes and the IP degrading oxidized calmodulin faster than the SP (Fig. 3b,c).

We confirmed this observation when digesting hemoglobin with purified 20S proteasomes. Here, we followed the degradation of ^3^H-labeled hemoglobin by measuring the release of small radioactive peptides, resistant to trichloroacetic acid precipitation (Fig. 3d,e). Like oxidized calmodulin, oxidized hemoglobin was more rapidly degraded by β5i­-containing 20S proteasomes (Fig. 3e). In this case, however, we also observed a partial degradation of unoxidized hemoglobin by 20S proteasomes (Fig. 3d), probably because hemoglobin is highly susceptible to spontaneous oxidation, through conversion of the ferrous form (Fe^2+^) of the heme to its ferric form (Fe^3+^) leading to reactive oxygen species production and autoxidation of the protein^47^. Overall, our results suggest that IP and intermediate 20S proteasomes more rapidly degrade oxidized proteins.

Because some reports described degradation of non-ubiquitinated proteins by the 26S proteasome^48, 49^, we also purified 26S proteasomes from the four 293 lines and monitored their ability to degrade oxidized calmodulin (Fig. S6a–c). After 8 h of digestion, we observed no degradation of oxidized calmodulin by any of the 26S proteasome subtypes (Fig. S6c), despite their high activity on fluorogenic substrates (Fig. S6b). In the same conditions, 20S DIP efficiently degraded oxidized calmodulin (Fig. S6c right panel). These results support our conclusion that ubiquitin-independent degradation of oxidized proteins is a selective attribute of 20S proteasomes.

### Alteration of protein structure targets oxidized proteins to the 20S proteasome

We next investigated whether the preferential targeting of oxidized proteins to the 20S proteasome resulted from the modification of protein conformation. In particular, the exposure of hydrophobic patches by oxidized proteins was suggested to facilitate opening of the α-gate of the 20S proteasome to favor degradation of oxidized proteins^50^.

**Figure 4:**
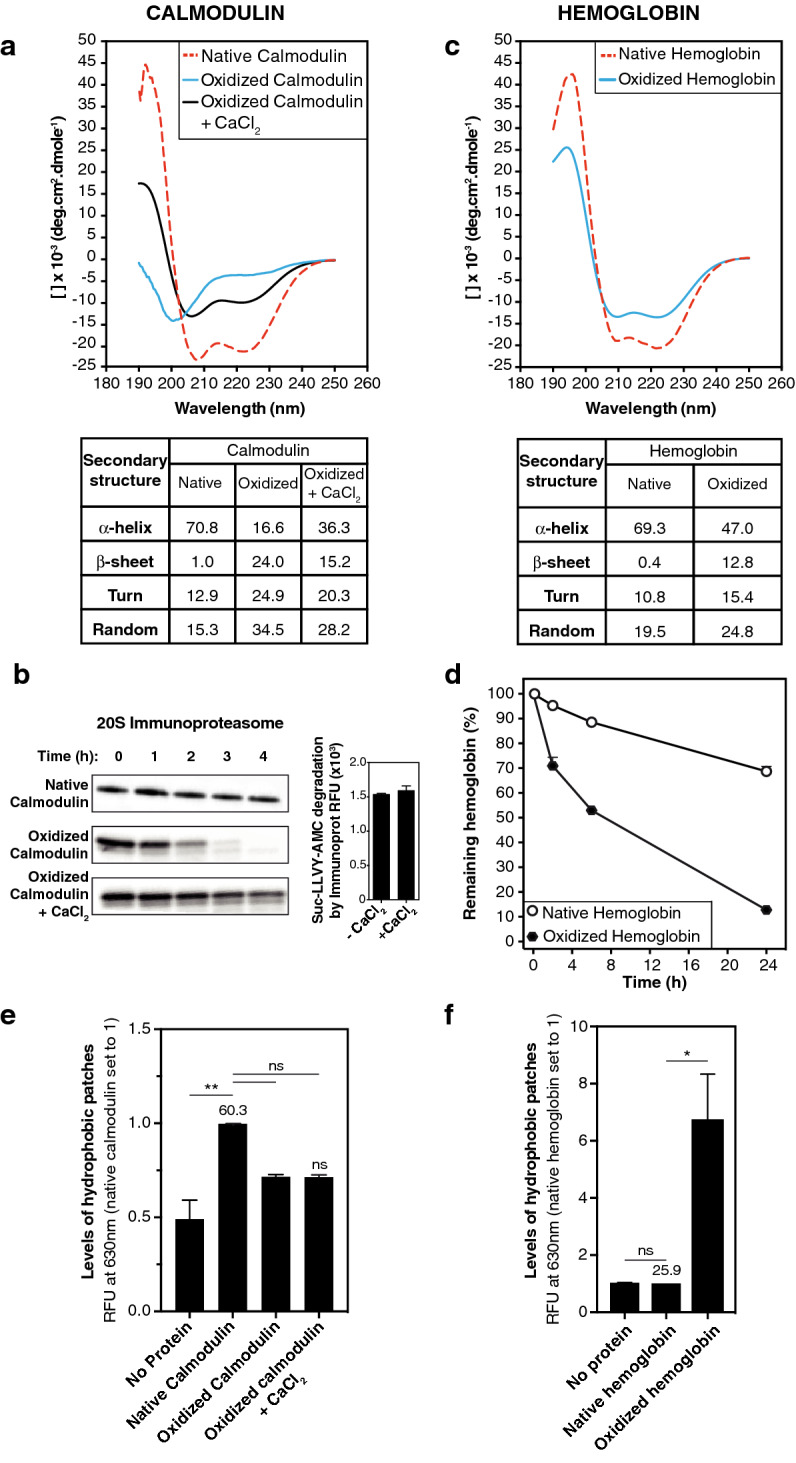
Degradation of oxidized proteins is triggered by disruption of their structure. (**a**) Analysis by circular dichroism of the secondary structure of native ^52, 53^calmodulin, oxidized calmodulin and oxidized calmodulin treated with 0.5 mM CaCl_2_. The top panel shows the circular dichroism spectra and the bottom panel shows percentages of the different secondary structures obtained by analyzing the circular dichroism spectra using the Contin-LL program. (**b**) The left panel illustrates a western blot showing that CaCl_2_ treatment of oxidized calmodulin reduced its degradation by 20S immunoproteasomes. These results are representative of three independent experiments. The right panel shows that CaCl_2_ did not affect the activity of 20S immunoproteasomes, as measured with fluorogenic substrate Suc-LLVY-AMC. (**c**) Analysis by circular dichroism of the secondary structure of native hemoglobin and oxidized hemoglobin. The top panel shows the circular dichroism spectra and the bottom panel shows percentages of the different secondary structures. (**d**) Kinetics of degradation of labeled unoxidized and oxidized hemoglobin. These two forms of hemoglobin were incubated with the 20S IP, and protein degradation was monitored as described in Fig. 3e. Similar results were obtained from seven independent experiments. (**e**, **f**) Assessing the level of surface exposed hydrophobic patches in the three forms of calmodulin (**e**) and in the two forms of hemoglobin (**f**) using the Nile Red probe. Nile Red was incubated with the different samples and its fluorescence at 630 nm, which increases in a non-polar environment, was measured. Relative fluorescence units (RFU) at 630 nm of each sample was reported to the RFU at 630 nm of the corresponding native protein, and the absolute RFU values at 630 nm of the native proteins are indicated on the histogram. All values are mean + SEM of three independent experiments. Full-length images for (b) are presented in Fig. S12.

Using circular dichroism analysis, we confirmed that oxidation of calmodulin induced a dramatic change in secondary structure (Fig. 4a), which correlated with an increased degradation by the 20S proteasome (Fig. 4b left panel). Moreover, the addition of calcium chloride to oxidized calmodulin partially restored its secondary structure (Fig. 4a) and decreased its degradation by the IP, whose intrinsic activity was not affected by the addition of calcium ions (Fig. 4b). These results confirmed the observations of Ferrington et al*.*^51^ and suggested that the loss of protein structure and not the presence of oxidized residues targets oxidized proteins to the catalytic chamber of the 20S proteasomes. Accordingly, when we analyzed the secondary structure of oxidized and native hemoglobin by circular dichroism, we also observed a disruption of secondary structure of oxidized hemoglobin that correlated with increased degradation by the 20S IP (Fig. 4c,d).

Oxidized hemoglobin also exposed more hydrophobic patches, as measured with Nile Red (Fig. 4f). However, hydrophobic patches did not seem involved in targeting oxidized proteins to 20S proteasomes since calmodulin oxidation actually decreased exposure of hydrophobic patches, and CaCl_2_ did not affect the level of exposed hydrophobic patches (Fig. 4e). We therefore conclude that disruption of protein structure is the main factor that targets oxidized proteins to the 20S proteasome.

### Degradation rate is independent of the presence of methionine sulfoxides

**Figure 5:**
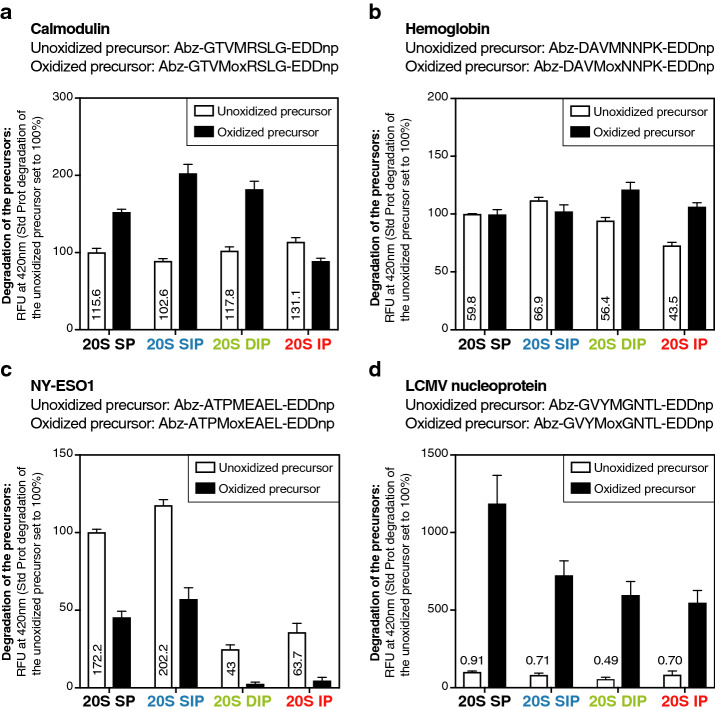
The presence of oxidized residues does not explain the differential degradation of oxidized proteins by the four 20S proteasome subtypes. Assay comparing the fluorescence emitted following the degradation of oxidized and unoxidized FRET (Fluorescence resonance energy transfer)-peptides by the four proteasome subtypes. Oxidized and unoxidized precursor peptides were derived from (**a**) calmodulin, (**b**) hemoglobin, (**c**) NY-ESO1 and (**d**) LCMV nucleoprotein. Unoxidized precursor peptides contained a methionine residue which was replaced by methionine sulfoxide (Mox) in the corresponding oxidized precursor. These precursor peptides were incubated with the four proteasome subtypes, and their degradation was monitored by measuring fluorescence at 420 nm. RFU at 420 nm of each sample was reported to the RFU at 420 nm of the unoxidized precursor degraded by the SP. The absolute RFU values at 420 nm of the unoxidized precursors degraded by the four proteasome subtypes are indicated on the histogram. All values are mean + SEM of three independent experiments.

Even though oxidized residues do not target oxidized proteins to the proteasome, their presence could explain the better degradation of oxidized proteins by β5i-containing 20S proteasomes. To test this hypothesis, we generated four 8-amino acid long precursor peptides corresponding to sequences found either in calmodulin, in the β-chain of hemoglobin or in antigenic proteins such as NY-­ESO­1^54^ and the LCMV nucleoprotein^55^. All four precursor peptides contained either a methionine residue (unoxidized precursor) or a methionine sulfoxide at the corresponding position (oxidized precursor). Each precursor peptide also had its C-­terminal residue coupled to a quencher (EDDnp) that absorbs the fluorescent signal emitted by the fluorescent group (Abz) bound to the N-terminus. Upon peptide cleavage, the quencher is released from the fluorescent probe and fluorescence can be detected (Fig. 5). Comparing the degradation of unoxidized versus oxidized precursors, we observed that the presence of oxidized methionine either increased (Fig. 5a,d), reduced (Fig. 5c) or did not modify (Fig. 5b) cleavage of the fluorogenic peptides by the four proteasome subtypes. Interestingly, the hierarchy of cleavage of the oxidized peptides by the four proteasome subtypes did not reflect the one observed for the degradation of oxidized proteins: the SP was even in some cases more efficient than the other subtypes (Fig. 5c,d). We therefore conclude that the presence of oxidized methionines inside the substrate protein does not explain the differential degradation of oxidized proteins by the four proteasome subtypes.

### The β5i catalytic subunit is crucial for degradation of oxidized proteins

**Figure 6:**
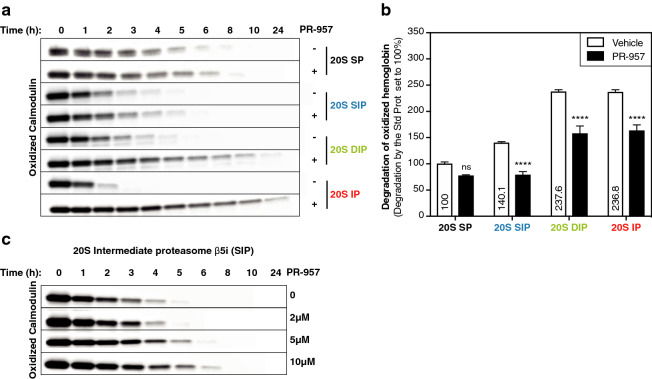
Catalytic immuno-subunit β5i plays an important role in the degradation of oxidized proteins by β5i-containing 20S proteasomes. (**a**) Western blot analysis of the kinetics of degradation of oxidized calmodulin by the four proteasome subtypes in the presence or absence of the β5i-specific inhibitor PR-957 (1 μM). These results are representative of three independent experiments. (**b**) Assessing the degradation of the radiolabeled oxidized hemoglobin by the four proteasome subtypes in the presence or in the absence of the β5i-specific inhibitor PR-957 after 24 h of digestion. The percentage of degraded hemoglobin was reported in each case to the percentage of degraded hemoglobin by the SP in the absence of PR-957, and the absolute percentages of degraded hemoglobin in the vehicle condition are indicated on the histogram. Error bars are SEM of three independent experiments. (**c**) Western blot analysis of the kinetics of degradation of oxidized calmodulin by the intermediate proteasome β5i treated with increased concentrations of the PR-957 inhibitor. These results are representative of three independent experiments. Full-length images for (a) and (c) are presented in Fig. S13.

We then tested whether the catalytic subunits present in 20S IP and intermediate proteasomes could explain their increased ability to degrade oxidized proteins. Since they share the β5i catalytic subunit, we hypothesized that this subunit might be responsible. To test this, we digested oxidized calmodulin and hemoglobin with purified 20S proteasomes, in the presence of the β5i-specific inhibitor PR-­957. The concentration of PR-957 was chosen to block the chymotrypsin-like activity of the IP and intermediate proteasomes without affecting that of the SP. Indeed, at higher concentrations, PR-957 is known to display off-target activity on the β5 subunit (Fig. S7)^56^. We observed that PR­-957 slowed down the degradation of oxidized calmodulin and hemoglobin by both IP and DIP (Fig. 6a,b). Degradation by SIP was also affected by PR­-957 (Fig. 6a,b), but to a lesser extent for oxidized calmodulin (Fig. 6a). Increasing the concentration of PR-­957 up to 10 μM slightly improved the inhibition of oxidized calmodulin degradation by SIP (Fig. 6c). To ascertain that the effect of PR-­957 resulted from β5i inhibition and not β1i, we repeated digestions with IP and DIP in the presence of the β1i-specific inhibitor calpeptin. Calpeptin did not block the degradation of oxidized calmodulin by IP and DIP at concentrations that inhibited hydrolysis of the β1i-specific fluorogenic substrate (Fig. S8). All in all, in β5i-containing 20S proteasomes, the catalytic activity of subunit β5i plays a critical role in the degradation of oxidized proteins.

### Degradation of intrinsically disordered protein tau by β5i-containing 20S proteasomes

**Figure 7:**
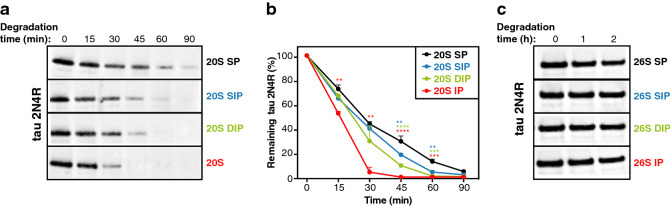
More efficient degradation of intrinsically disordered protein tau by 20S proteasomes containing subunit β5i. (**a**) Western blot analysis of the kinetics of degradation of recombinant tau 2N4R by the four 20S proteasome subtypes. (**b**) Densitometric evaluation of the kinetics of the degradation of tau by the four 20S proteasome subtypes. All values (+ SEM) are collected from three independent experiments. (**c**) Western blot analysis of the kinetics of degradation of tau by the four 26S proteasome subtypes. These results are representative of three independent experiments. Full-length images for (a) and (c) are presented in Fig. S14.

Our observation that ubiquitin-independent degradation of oxidized proteins by 20S proteasomes was triggered by disruption of their structure prompted us to investigate whether intrinsically disordered proteins were also degraded by 20S proteasomes. A few reports support the contention that such proteins can be degraded by 20S proteasomes in an ubiquitin-independent manner^57–59^. It is the case of tau, a protein that accumulates in Alzheimer disease and exhibits little or no secondary structure. We compared the degradation of recombinant tau incubated with purifed 20S or 26S proteasomes. We observed that 20S proteasomes degraded tau (Fig. 7a,b), while 26S proteasomes did not (Fig. 7c). This result confirmed that tau was degraded in an ATP-ubiquitin-independent manner^59^, and suggested that the disordered structure of tau targeted this protein for degradation by 20S proteasomes, in a manner analogous to oxidized proteins. Accordingly, we observed that, like oxidized proteins, tau was degraded faster by β5i­-containing 20S proteasomes. Overall, these results support a model in which proteins that are disordered, either intrinsically or as a result of oxidation, are efficiently degraded in an ubiquitin-independent manner by the 20S proteasomes that contain subunit β5i, i.e. the 20S immunoproteasome and the two 20S intermediate proteasomes.

## Discussion

The proteasome is key to maintaining cell homeostasis and establishing the MHC class I immune repertoire. In particular, the immunoproteasome (IP), which is expressed in immune cells and induced under inflammatory conditions, is essential for the production of a high affinity MHC class I repertoire^15, 17, 18^. In addition to the standard proteasome (SP) and the IP, two additional proteasome subtypes [intermediate proteasomes β5i (SIP) and β1i–β5i (DIP)], containing a mix of constitutive and inducible subunits, are found in healthy tissues (such as kidney, small bowel or liver) and in tumors^6, 60^. Although these intermediate proteasomes constitute around 30–50% of the total proteasome content found in healthy tissues, their specific function, aside from altering the antigenic peptide repertoire, was not yet elucidated. We therefore studied the potential role of intermediate proteasomes in protein homeostasis notably in the degradation of ubiquitinated and oxidatively damaged proteins.

The controversy regarding better clearance of ubiquitinated proteins by the IP and the lack of insights about the role of the intermediate proteasomes in this process, led us to compare the four proteasome subtypes for their ability to degrade ubiquitinated proteins. We used a system composed of four cell lines of the same origin that only differ by their proteasome content. We used them to compare the four proteasome subtypes for their ability to clear the pool of ubiquitinated proteins. We observed no difference in the clearance of total ubiquitinated proteins in the four cell lines. However, the interpretation of this result was complicated by the involvement of deubiquitinases, which also play an important role in the removal of ubiquitinated proteins. We therefore studied the degradation of two specific substrates, p21 and c-myc, for which we first ascertained that the degradation was ubiquitin and proteasome-dependent. In particular, the decay of p21 and c-myc was fully prevented by inhibition of ubiquitination or proteasome activity. We monitored the degradation of these substrates by western blot using specific antibodies, bypassing thereby any involvement of deubiquitinases. We observed no difference in the degradation of p21 and c-myc in the four cell lines. By showing that the SP and IP did not differ in their capacity to degrade ubiquitinated proteins, our results differ from those published by Seifert et al.^21^, but confirm those obtained by Nathan et al.^23^. Our results also show that intermediate proteasomes SIP and DIP degrade ubiquitinated proteins with the same efficiency as SP and IP. Previous studies showed that the rate of degradation by the 26S proteasome was limited by the 19S RP^61–63^, which controls recognition and removal of ubiquitin moieties, unfolding of the protein substrate and its progression into the 20S core. This may explain why the difference in catalytic subunits of the proteasome subtypes does not affect the degradation of ubiquitinated proteins, as their impact is likely masked by the rate-limiting effect of the 19S RP. This implies, however, that the 19S RP functions similarly in the four proteasome subtypes. This is supported by crystallographic studies of SP and IP, which revealed a high structural similarity between their outer α-rings, which comprise the binding interface between the 19S RP and the 20S proteasome^12^. Moreover, no difference in 19S subunit content was observed between the four proteasome subtypes^64^. In vivo cross-linking experiments performed on the four 293 cell lines, followed by LC–MS/MS analysis of immunopurified proteasomes, confirmed that the amount of 19S RP bound to 20S proteasomes was similar for all four proteasome subtypes^64^. Taken together, our results combined with previous studies suggest that the lack of difference in the degradation of 26S substrates by the four proteasome subtypes is caused by a similar functioning of the 19S RP in the four proteasome subtypes.

Although in normal conditions the 26S proteasome is responsible for the degradation of most cellular proteins^3^, its role in degrading oxidized proteins that can accumulate under oxidative stress conditions is unclear. Contradictory results regarding the protective role of the 26S proteasome under oxidative stress conditions have made the field extremely controversial. On the one hand, several studies suggested that degradation of oxidized proteins was ensured by the 26S proteasome^65–67^, showing that cells with compromised 26S proteasomes accumulated oxidized proteins under oxidative stress, and that increasing 26S proteasome activity by blocking deubiquitinase Usp14 increased the degradation of oxidized proteins under oxidative stress^65, 67^. Moreover, Manohar et al*.* showed that after oxidative stress, half of the oxidized proteins were conjugated with K48 ubiquitin, which targets them for proteasomal degradation^66^. On the other hand, other studies suggested that degradation of oxidized proteins was mediated by 20S more than 26S proteasomes^25, 27, 40–42, 68–70^. They showed that oxidized proteins were better degraded by 20S proteasomes^25, 68^, and that cells with compromised 26S proteasomes or with a compromised ubiquitinated system were not more sensitive to oxidative stress than wild type cells^27, 70^. Moreover, they showed that the ubiquitination system was sensitive to oxidative stress^69^ and that the 26S proteasome disassembled under oxidative conditions with the help of ECM29, thus increasing the abundance of 20S proteasomes^41, 42^. Finally, the 20S role in degradation of oxidized proteins was further supported by studies suggesting that oxidative stress was associated with depletion of ATP, which is needed for 26S proteasome functioning^71–73^.

As a first step, we evaluated the effect of oxidative stress on proteasome composition. Our mass spectrometry analysis of regulators bound to 20S proteasomes confirmed an increased proportion of free 20S proteasome in oxidant-damaged cells. This increased abundance of 20S correlated with a decreased level of 26S and with a recruitment of ECM29, which triggers disassembly of the 26S proteasome^41, 42^. This confirms the observations by Wang et al*.* and suggests that the 26S proteasome is sensitive to oxidative stress because it partially dissociates into 20S proteasome^41, 42^. Because degradation of oxidized proteins is possibly independent of the 26S proteasome and the ubiquitin system, we separated the study of degradation of oxidized proteins from that of ubiquitinated proteins. Because in our hands degradation of oxidized proteins could not be reliably assessed in cells, we analyzed their ubiquitin-independent degradation in vitro using the oxidant-resistant 20S proteasomes. Our in vitro experiments confirmed that 26S proteasomes could not degrade oxidized proteins in an ubiquitin-independent manner, while 20S proteasomes could. When comparing the in vitro degradation of oxidized calmodulin and hemoglobin by the four 20S proteasome subtypes, we observed that their turnover was faster with the IP and the two intermediate proteasomes when compared to the SP. Our data suggest that, aside from its role in the generation of MHC class I-restricted peptides, one of the functions of the IP might be to degrade oxidized proteins induced in inflammatory conditions, i.e. when IP expression is induced. Because SIP and DIP are both expressed in healthy tissues in the absence of obvious inflammation^6, 60^, their higher propensity to degrade oxidized proteins suggests that they could protect healthy tissues from the accumulation of oxidized proteins in non-inflammatory oxidative stress conditions, in which IP is not induced. The remaining 26S proteasomes that did not dissociate in oxidative stress could also contribute to the elimination of oxidized proteins, but these proteins should then be ubiquitinated and therefore equally degraded by the four 26S proteasome subtypes, because of the rate-limiting effect of the 19S RP, as discussed above. The absence of 19S RP in 20S proteasomes eliminates this effect, thereby unmasking the effect of the catalytic subunits of the proteasome subtypes. In sum, while oxidized proteins that are ubiquitinated are expected to be similarily degraded by the four 26S proteasome subtypes, oxidized proteins that are eliminated in an ubiquitin and ATP-independent manner by 20S proteasomes appear to be better degraded by IP and the intermediate proteasomes than by the standard proteasome.

Structural studies performed on 20S proteasomes reveal that the central pores formed by the α-­rings are obstructed by the N-­termini of their α-­subunits^74^. The gates of our purified 20S proteasomes were predominantly closed (Fig. S5c). Closed-gated proteasomes restrict the entry of proteins to their catalytic chambers. The opening of their gates requires the help of the different regulatory particles, or can be induced in vitro using hydrophobic peptides or using low concentrations of SDS^74, 75^. Our in vitro assays proved that oxidized proteins were able to enter the catalytic chamber of closed-gated 20S proteasomes without the help of any regulatory particle or any gate-opening treatment, while native proteins were unable to do so. This unregulated entry of oxidized proteins into 20S proteasomes led us to evaluate whether a change in protein structure could explain the targeting of oxidized proteins for degradation by 20S proteasomes. Our circular dichroism data clearly showed that oxidation caused drastic modifications in the secondary structure of calmodulin and hemoglobin. Moreover, using Ca^2^^+^ to restore the structure of oxidized calmodulin, we confirmed that the entry into the closed 20S proteasome was triggered by the disrupted structure of oxidized proteins rather than the increased exposure of hydrophobic patches or the presence of oxidized residues. This notion that the lack of structure targets proteins for degradation by the 20S proteasome was fully confirmed when we tested degradation of tau, an intrinsically disordered protein. We found that 20S proteasomes efficiently degraded tau in an ubiquitin-independent manner, while 26S proteasomes did not. Although our circular dichroism data show that degradation of oxidized proteins by 20S proteasomes correlates with the secondary structure alteration, it is unclear whether disruption of only the tertiary structure would suffice for ubiquitin-independent degradation by 20S proteasomes. In future studies, it will be interesting to determine the exact role of secondary and tertiary structure modifications in the unregulated degradation by 20S proteasomes.

Our study also revealed that the degradation of oxidized proteins was dependent on the nature of the catalytic subunits of 20S proteasomes. Using the β5i-specific inhibitor PR­-957, we showed that the β5i catalytic subunit was essential for the degradation of oxidized calmodulin and hemoglobin. Interestingly, β5i was the main subunit involved in the degradation of oxidized calmodulin by DIP and IP. This result contrasts with the notion that all catalytic subunits contribute to the degradation of proteins by the 26S proteasome^76^. This divergence likely reflects the fact that the ubiquitin-dependent degradation of 26S protein substrates is different from the ubiquitin-independent degradation of oxidized proteins by the 20S proteasome. Unlike 26S protein substrates, which are unfolded by the 19S RP before entering the catalytic chamber, oxidized proteins probably access the catalytic chamber of the 20S proteasome through their disordered regions, which we found to be essential for degradation by 20S proteasomes. The improved degradation of oxidized proteins in the presence of β5i could relate to an increased sensitivity of disordered regions to the β5i catalytic activity. Another possibility could be that disordered regions more readily access the β5i catalytic site through narrow side openings at the interface between the α and β-rings, which were previously suggested to allow the entry of disordered proteins and polypeptides^4^. This proposed functional link between disordered protein regions and β5i catalytic activity is strikingly supported by our observation that tau, an intrinsically disordered protein, was better degraded by β5i-containing 20S proteasomes, even though it was not oxidized.

Although our results showed that the β5i catalytic activity was central to the degradation of oxidized proteins by 20S proteasomes, we observed that IP was more efficient than DIP at degrading oxidized proteins. This suggests that the β2i catalytic subunit, which is exclusively present in IP, might also be involved. The presence of β2i could slightly alter the proteasome structure, rendering β5i more active. On the other hand, degradation of oxidized proteins by SIP was less sensitive to the β5i inhibitor PR-­957 when compared to IP or DIP. This suggests that, in SIP, either β5i is not the only subunit involved in degradation of oxidized proteins, or the presence of β1 in place of β1i (which is found in both IP and DIP) alters the structure of β5i preventing full inhibition by PR-957. The latter hypothesis is supported by the fact that the PR-957 dose required to fully block the chymotrypsin-like activity of SIP is higher than for IP and DIP (Fig. S7). Solving the crystal structure of the four proteasome subtypes bound to PR-957 should help to understand this differential effect of the inhibitor.

Besides confirming the partial dissociation of 26S proteasome upon oxidative stress, our mass spectrometry approach, which relied on affinity purification with an anti-α2 antibody, allowed us to have a broad view of all proteins associated with 20S proteasomes. We observed a significant recruitment of the PA28γ and the IFNγ-induced PA28α/β regulators, highlighting their potential function in oxidative stress (Fig. 2). Because the increased binding of PA28α/β and PA28γ to the 20S proteasome did not correlate with an increased expression in the total lysate (Fig. S9), these regulators seem to be actively recruited to the 20S proteasome upon oxidative stress. An activator function was already inferred for PA28α/β in the degradation of oxidized proteins^77–79^. Since PA28α/β is expressed, like IP, in inflammatory conditions, its increased binding to proteasomes upon oxidative stress could reinforce the activity of the IP towards oxidized proteins. In future studies, it will be interesting to explore whether proteasomes associated to PA28γ are more efficient than 20S proteasomes in degrading oxidized proteins, and to investigate how this regulatory particle could affect the function of the four proteasome subtypes.

In conclusion, we show here that the SP, the IP and the intermediate proteasomes all degrade ubiquitinated proteins at similar rates. On the other hand, we confirm that oxidative stress partially dissociates the 19S regulator from the 26S proteasome, resulting in more 20S in the cell. 20S proteasomes can degrade oxidized -but not native- proteins and this is triggered by secondary structure alterations that occur during oxidation. Accordingly, 20S proteasomes also degrade intrinsically disordered proteins, like tau. Catalytic subunit β5i is key to the turnover of oxidized and disordered proteins, which are both better degraded by 20S IP, DIP and SIP as compared to 20S SP. Therefore, β5i-containing proteasomes seem to play a key role in the clearance of disordered and oxidatively damaged proteins.

## Materials and methods

### Degradation of ubiquitinated proteins

Four HEK293-EBNA cell lines, each expressing one proteasome subtype^6^, were used to study the kinetics of degradation of ubiquitinated p21 and c-myc. For the kinetic analysis, 1 × 10^6^ cells of the four 293 lines were treated with 50 μg/ml of cycloheximide (Cell Signaling) and collected for lysis every hour and this for 5 h.

To confirm that p21 and c-myc were degraded in a proteasome and ubiquitin-dependent manner, 1 × 10^6^ 293 cells expressing standard proteasome were treated either with DMSO, 20 μM of MG132 (Selleck Chemicals), 5 μM of MLN7243 (Active Biochem) or 1 μM of Bortezomib (Santa-Cruz sc-217785) for 30 min. Cycloheximide was then added at a concentration of 50 μg/ml for 5 h.

Cells were then collected and lysed in lysis buffer containing 0.1% SDS, 1% deoxycholic acid, 0.5% NP40 supplemented with protease and phosphatase inhibitors (Roche). Proteins were quantified using the BCA protein assay (from Thermo Fisher Scientific) and analyzed by western blot.

### Induction of oxidative stress in 293 cells and in vivo cross-linking

1 × 10^8^ 293 SP cells were treated or not with 2 mM of H_2_O_2_ for 30 min. They were then cross-linked for 15 min with 0.1% of formaldehyde and the cross-linking reaction was finally quenched using 125 mM of glycine. Cells were washed three times with PBS and then lysed in lysis buffer (10 mM Hepes pH 7.9, 10 mM KCl, 5 mM MgCl_2_, 10% glycerol, 10 mM ATP, 1% NP-40, protease and phosphatase inhibitors; Roche) for 15 min at 4 °C. The lysate was sonicated, centrifuged and the protein concentration was quantified using a detergent-compatible assay (DC assay; Bio-Rad).

### Immuno-purification of proteasomes and LC–MS/MS analysis

Immuno-purification of the proteasomes from the in vivo cross-linked lysates was performed as previously described^44^. Briefly, proteasomes were purified by incubating the lysates with 100 mg of CNBr sepharose beads (GE Healthcare) covalently-bound to 0.8 mg of the antibody specific for the α2 subunit of the proteasome (MCP21). The supernatant was collected, and the beads were washed three times with 40 bead volumes of washing buffer (20 mM Tris–HCl pH 7.6, 1 mM EDTA, 10% glycerol, 150 mM NaCl, 0.1% NP-40, 2 mM ATP and 5 mM MgCl_2_). Finally, proteins were eluted with 0.5 ml of elution buffer (20 mM Tris–HCl pH 7.6, 1 mM EDTA, 10% glycerol, 3 M NaCl, 2 mM ATP and 5 mM MgCl_2_). Two additional cycles of purification were conducted using the collected supernatant. All fractions were pooled. 20S proteasome quantification by sandwich ELISA assay was performed as previously described^44^.

LC–MS/MS analysis was performed as previously described^43, 45^. Briefly, immuno-purified proteasome samples were precipitated with 20% TCA, washed with acetone and then denatured by boiling at 95 °C for 30 min in the Laemmli buffer. Proteins were alkylated and concentrated on 12% acrylamide SDS-PAGE gel as a single band, which was cut and washed. Trypsin digestion was then performed overnight at 37 °C and the peptides were extracted from the gel. The digestion mixture was then dried in a Speed-Vac and resuspended with 2% acetonitrile, 0.05% trifluoroacetic acid. 5 μL of each peptide sample corresponding to 2.5 μg of 20S proteasome (estimated by ELISA) were then analyzed by nano-LC–MS/MS using an UltiMate3000 system (Dionex) coupled to LTQ-Orbitrap Velos mass spectrometers (Thermo Fisher Scientific). The Mascot Daemon software (version 2.3.2; Matrix Science, London, UK) was used for database search. Protein identification and validation were performed as previously published^43, 45^.

### Relative quantification of free 20S proteasome and 20S associated regulators

In order to quantify free 20S proteasome and proteasome-bound regulators, we used a mass spectrometry-based approach as previously described^45^. For each of the subunits of the complex, a Protein Abundance Index (PAI) was calculated, which is defined as the average of extracts ion chromatograms (XIC) area values corresponding to the three most intense reference tryptic peptides identified from the protein. If only one or two peptides were identified (for example in the case of low abundance proteins), then the PAI was calculated on the basis of these two XIC area values. All PAIs were normalized (NPAIs) with the total amount of 20S proteasome, which was defined as the mean of the PAIs of 20S proteasome non-catalytic subunits (subunits α1 to α7, β3, β4, and β7) divided by two because each core proteasome subunit is integrated twice in each 20S core complex.

Five types of complexes were considered: the free 20S complex, the 19S-associated, the PA28αβ-associated, the PA28γ-associated, and the PA200-associated proteasomes. The 19S-associated proteasome fraction was calculated as the mean of the normalized NPAIs of the 19S subunits. The fraction of 20S proteasome in association with PA28αβ was calculated from the normalized NPAIs of each PA28α and PA28β subunits, taking into account the stoichiometry of both subunits in the final α4β3 heptameric complex. Similarly, for the PA28γ-associated 20S proteasome, the homo-heptameric structure of the PA28γ regulator complex was taken into account. The fraction of 20S proteasome that interacts with PA200 was obtained directly from the normalized NPAI of this large monomeric protein. As each regulator can be associated with the 20S complex in a stoichiometry of one or two regulators per 20S core, we approximated the fraction of regulator-associated proteasome by considering that the overall (20S proteasome: regulator) stoichiometry was (1:1.5). Thus, the fractions obtained were divided by 1.5 to take this stoichiometry into account. Using this approach, hybrid forms of the proteasome, corresponding to a 20S proteasome core interacting with one type of regulator, such as 19S, on one side, and another type of regulator, such as PA28, on the other side, could not be considered. Finally, the free 20S proteasome fraction was considered to correspond to the remaining 20S entities, by subtracting the regulator-associated 20S forms from the total 20S complexes^45^.

### Purification of the four 20S proteasome subtypes

Purification of 20S proteasome was performed as previously described^31^ using frozen pellets of 1 × 10^9^ 293 cells. Cell pellets were lysed using 20 ml of buffer A (100 mM KCl, 5 mM MgCl_2_, 10 mM HEPES pH 7.2) supplemented with 0.1% NP-40. The suspension was passed 3 times through a 23-gauge needle then 2 times through a 25-gauge needle and finally through a 27-gauge needle. The lysate was centrifuged at 30,000 g for 30 min at 4 °C with the SW32Ti rotor and the supernatant loaded into 10 ml packed volume of DEAE-Sephacel (Amersham Pharmacia Biotech). The column was washed using 5 bead volumes of buffer A and beads were eluted in 500 mM KCl, 5 mM MgCl_2_, 10 mM Hepes, pH 7.2. All proteins that were bound to the column were collected and precipitated with 35% of ammonium sulfate overnight under agitation at 4 °C. The precipitated proteins were discarded by centrifugation at 17,000 g for 20 min at 4 °C and the supernatant was loaded onto a phenyl-Sepharose column. Elution was performed with a gradient of 35–0% of ammonium sulfate in buffer A. Fractions of 1 ml were collected, analyzed by sandwich ELISA as previously described^80^ and positive fractions containing the proteasome were pooled and precipitated with 80% ammonium sulfate overnight under agitation at 4 °C. Proteins that precipitated were collected by centrifugation at 12,000 g for 10 min at 4 °C, resuspended with 4 ml of buffer A and loaded into a gradient of 15–40% of sucrose in buffer A. The gradient was centrifuged at 29,200 rpm for 36 h at 4 °C using the rotor SW32Ti. Fractions of 0.8 ml were collected, tested by sandwich ELISA to identify the fractions containing the proteasome, pooled and diluted 5 times in buffer A. Pooled fractions were then concentrated using the Vivaspin 6 (cutoff 10,000 Da). A volume of 2 ml was collected and injected into a Superose-6 10–300 GL column. Purified proteasomes were collected from this size-exclusion column at 11–13 ml and then concentrated using the Vivaspin 6. Proteasome concentration was measured by performing three independent sandwich ELISA and the proteasome purity was assessed by migration in a PAGE gel and silver staining as well as by liquid chromatography coupled to LC–MS/MS. Proteasome activity was tested using the fluorogenic substrates Suc-LLVY-AMC, Z-LLE-AMC or Boc-LRR-AMC (Enzo Life Sciences). Each of the four proteasome subtypes was purified independently three times and the in vitro kinetic assays were performed at least twice with each batch of proteasome.

### Oxidation of calmodulin with H_2_O_2_

Recombinant bovine calmodulin (Merck Millipore Cat#14-368) or calmodulin purified from bovine brain (Merck Millipore Cat#208690-1MG) were resuspended in 20 mM of Tris–HCl pH 7.0 to a concentration of 2 mg/ml (similar results were obtained with both calmodulin sources). Half of the resuspended protein was oxidized by incubating 1 mg/ml of calmodulin with 50 mM Pipes pH 6.2, 100 mM KCl, 0.1 mM CaCl_2_, 1 mM MgCl_2_, in the presence of 50 mM H_2_O_2_ at room temperature for 24 h. Oxidized calmodulin was then dialyzed 3 times against 20 mM Tris–HCl, pH 7.0 and its concentration was measured using BCA protein assay (from Thermo Fisher Scientific). Oxidation of the protein was verified by separating native and oxidized calmodulin (1.2 μg) in a denaturing 12% polyacrylamide gel, which was stained with Page blue protein staining solution (Thermo Fisher Scientific). As expected, the oxidized calmodulin migrated slower than its native counterpart demonstrating that the protein was efficiently oxidized (Fig. S11). Further verification was performed by analyzing the native and the oxidized calmodulin by HPLC–MS. This showed that more than 80% of the calmodulin bore 9 methionine sulfoxides and the remaining 20% contained at least 6 methionine sulfoxides.

### Degradation of calmodulin by the 20S proteasome

Native or oxidized Calmodulin (2.9 μg) were mixed with purified 20S proteasomes (2.1 μg) in a buffer containing 5 mM Tris–HCl pH 7.4, 1 mM MgCl_2_, 10 mM KCl and 0.01 mM EGTA in a final volume of 40 μl and the reaction was incubated at 37 °C. Samples (1.6 μl) were collected at different time points and the reaction was stopped by freezing the samples on dry ice. These samples were then analyzed by western blot. Where indicated, the proteolysis buffer was supplemented with 0.5 mM of CaCl_2_ and incubated with the oxidized calmodulin for 30 min at 37 °C prior to the addition of the 20S immunoproteasome. In some experiments, PR-957 (S7172-5MG SelleckChem) was incubated with purified proteasome for 30 min at 37 °C prior to the addition of the oxidized calmodulin.

### Labeling and oxidation of bovine hemoglobin

Reductive methylation of bovine hemoglobin (Sigma-Aldrich Cat#H2500) was carried out in 1 ml reaction volumes containing 1 mg protein, 10 nmoles ^3^H formaldehyde (Isobio (Fleurus) 10 Ci/mmole Cat#ART-0311-1), 20 mM NaCNBH_3_ (Thermo Fisher Scientific Cat#44892) and 100 mM Hepes, pH 7.5, according to the previously described protocol by Jentoft et al*.*^81^. The mixture was incubated at room temperature for 24 h. The tritium-incorporation yield measured after 10% TCA precipitation varied between 7–20%. One-half of the sample was then removed and incubated with 50 mM H_2_O_2_ for 24 h, which caused discoloration of the light brown hemoglobin solution. The labeled hemoglobin treated or not with hydrogen peroxide was then dialyzed 3 times against 20 mM Tris–HCl, pH 7.5.

Samples (1.5 μg) of native or oxidized hemoglobin were separated in a denaturing 4–12% polyacrylamide gel and stained with the SilverQuest Silver Staining kit (Thermo Fisher Scientific Cat#LC6070). For each of the 2 conditions, a band corresponding to the 15 kDa hemoglobin of the two monomers was clearly visible and the intensities of the bands were similar.

### Degradation of labeled hemoglobin by the 20S proteasome

Labeled hemoglobin (1.5 μg) was mixed with purified 20S proteasome (2 μg) in a final volume of 20 μl of buffer containing 5 mM Tris.HCl pH 7.4, 1 mM MgCl_2_, 10 mM KCl and 0.01 mM EGTA. The final reaction was then incubated at 37 °C and samples (2 μl) were collected after 0, 2, 6 and 24 h. The reaction was stopped by adding 23 μl H_2_O, 25 μl BSA (40 mg/ml) and 800 μl ice-cold 10% trichloroacetic acid (TCA). After 30 min on ice, the supernatant was directly transferred to a counting vial. Radioactivity of the supernatant was measured in 12 ml scintillation cocktail (Ultima Gold, Perkin Elmer Cat#6013329) using a Beckman LS6000 IC liquid scintillation counter. Where indicated, 1 μM of PR-957 was added with the digestion mixture.

### Purification of the four 26S proteasome subtypes

The four 26S proteasomes were purified from the four 293 cell lines by the Ubl-affinity method^82^ following the instructions of the kit (rapid 26S purification Kit-L Cat# J4320). Frozen pellets of approximately 300 × 10^6^ cells of the four 293 cells were used for the purification. Protein content was measured using Bradford assay. The concentration of the purified 26S proteasomes was estimated by western blot densitometry study. The levels of α2 subunit and 20S subunits of the four 26S preparation was compared to the levels of the same subunits of a 20S proteasome preparation of known concentration.

### Degradation of tau by 20S proteasomes

Recombinant tau 2N4R (8.3 μg) (Anaspec Cat#AS-55556-50) was mixed with purified 20S proteasomes (2.1 μg) in a buffer containing 5 mM Tris–HCl pH 7.4, 1 mM MgCl_2_, 10 mM KCl and 0.01 mM EGTA in a final volume of 40 μl and the reaction was incubated at 37 °C. Samples (1.6 μl) were collected at different time points and the reaction was stopped by freezing the samples on dry ice. These samples were then analyzed by western blot.

### Degradation of tau by 26S proteasomes

Recombinant tau 2N4R (1.35 μg) was mixed with purified 26S proteasomes (2.6 μg) in a buffer containing 5 mM Tris–HCl pH 7.4, 1 mM MgCl_2_, 10 mM KCl, 0.01 mM EGTA and 2 mM of ATP in a final volume of 20 μl and the reaction was incubated at 37 °C. Samples (1.6 μl) were collected at different time points and the reaction was stopped by freezing the samples on dry ice. These samples were then analyzed by western blot.

### Western blot

20 μg of protein lysates or samples collected from the degradation of calmodulin by the 20S proteasomes were separated on a denaturing 4–12% polyacrylamide gel and then transferred to a nitrocellulose (Thermo Fisher Scientific Cat#IB23001) membrane using the iBlot 2 transferring apparatus (Thermo Fisher Scientific). Membranes were blocked for 1 h at room temperature in PBS containing 5% of dry milk and 0.1% Tween 20, washed three times in the washing buffer (PBS, 0.1% Tween) and incubated with the primary antibody. The mouse antibody directed against calmodulin (Millipore Cat#05-173, RRID:AB_309644) was diluted at 1/5,000 and incubated 1 h at room temperature. The rabbit p21 antibody (Cell Signaling Technology Cat#2947S, RRID:AB_823586) and the rabbit c-myc antibody (Cell Signaling Technology Cat#13987, RRID: AB_2631168) were diluted at 1/1,000 and the mouse vinculin antibody (Sigma-Aldrich Cat#V9131,RRID_AB:477629) was diluted at 1/2,000. All these antibodies were diluted in the washing buffer supplemented with 5% of bovine serum and 0.02% azide. The rabbit polyclonal antibody B19 that recognizes all isoforms of tau^83, 84^ (kindly provided by Jean-Pierre Brion, Université libre de Bruxelles) was diluted at 1/2,000 in PBS supplemented with 2% BSA and 0.08% azide. Diluted antibodies were incubated with the membranes overnight under agitation at 4 °C. The membranes were washed three times with the washing buffer and then incubated with the secondary antibody for 1 h at room temperature under agitation. Goat anti-mouse HRP-conjugated IgG (R&D Systems Cat#HAF007, RRID:AB_357234) and goat anti-rabbit HRP-linked IgG (Cell Signaling Technology Cat#7074, RRID:AB_2099233) were both diluted 1/2,000 in the washing buffer supplemented with 5% of non-fat dry milk. Finally western blot signals were revealed with the chemiluminescent substrate West Pico SuperSignal (Thermo Fisher Scientific). Images were acquired by Fusion Fx camera from Vilber Lourmat. Quantification was performed with the Bio-1D (version 15.06b) software from Vilber Lourmat and with Image J.

### Proteasome activity in the presence of CaCl_2_

100 μM of the fluorogenic substrate Suc-LLVY-AMC were mixed with 10 nM of purified 20S immunoproteasome in a proteolysis buffer containing 5 mM Tris–HCl pH 7.4, 1 mM MgCl_2_, 10 mM KCl, 0.01 mM EGTA supplemented or not with 0.5 mM CaCl_2_. The final reaction volume (100 μL) was incubated at 37 °C for 2 h. The fluorescence intensity was measured for the digested peptides using the Glo-Max discover microplate reader (Promega Fitchburg) (Excitation 390 nm, Emission 415–445 nm).

### Circular dichroism

Native and oxidized calmodulin or hemoglobin were initially dissolved in 20 mM Tris–HCl pH 7.0 buffer. To study their circular dichroism spectra, the Tris–HCl buffer was replaced by 10 mM sodium phosphate buffer pH 7.0 by three rounds of dialysis. The concentration of the proteins were then evaluated using BCA protein assay (Thermo Fisher Scientific) and diluted in the phosphate buffer to reach a protein concentration of 0.2 mg/ml. Circular dichroism measurements were performed using a Jasco J-715 circular dichroism spectropolarimeter at 25 °C in a quartz cuvette with a 1-mm path length (#110-1-40; Hellma Analytics). Far UV spectra (190 to 250 nm) were recorded at a scan speed of 50 nm/min, 1 nm bandwith. Five spectra were measured and averaged. The percentage of α-helical and β-strand content was determined by sending the records to the Dichroweb server.

### Nile Red hydrophobicity assay

Nile Red (Sigma-Aldrich Cat#7385-67-3) was dissolved in DMSO to 0.25 mM. 0.2 μM of Nile Red was mixed in 40 mM HEPES–KOH pH 7.4 with either 10 μM of native or oxidized calmodulin or 5 μM of native or oxidized hemoglobin. Where indicated, oxidized calmodulin was pre-incubated 30 min with CaCl_2_. The fluorescence intensity was measured using the Glo-Max discover microplate reader (Promega Fitchburg) and measurement parameters were set to 520 nm excitation and 580–640 nm emission.

### Synthesis and degradation of the fluorescent precursors

The FRET-fluorescent precursor peptides, Abz-peptidyl-EDDnp, were synthesized by solid-phase peptide synthesis (SPPS) using conventional fluorenylmethoxycarbonyl (Fmoc) chemistry on a Symphony synthesizer (Protein Technologies Inc). Dnp Nova Tag resin was used as the support for peptide synthesis to couple the EDDnp (2,4-dinitrophenyl ethylenediamine) (NovaBiochem, Merck Cat#855053) quencher group to the C-terminal carboxyl residue of the peptide. Fmoc-Abz-OH (Bachem Cat#B-3260) was used as the building block to couple the N-terminal residue to the fluorescent group Abz (ortho-aminobenzoic). All of the peptides obtained were purified by reverse-phase HPLC to > 95% purity and characterized by mass spectrometry. Finally the lyophilized peptides were dissolved in DMSO to a concentration of 10 mM.

Unoxidized or oxidized precursors (100 μM) were mixed with purified 20S proteasomes (14 nM) in a proteolysis buffer containing 5 mM Tris–HCl pH 7.4, 1 mM MgCl_2_, 10 mM KCl and 0.01 mM EGTA. The final reaction (50 μL) was incubated at 37 °C for 2 h. The fluorescence intensity was measured using the SpectraMax 190 Microplate Reader (Excitation 320 nm, Emission 420 nm).

### Statistical method

For the analysis of the effect of oxidative stress on the proteasome-interacting proteins and for the analysis of the effect of the PR-957 inhibitor on the degradation of oxidized hemoglobin an unpaired student *t-*test was performed. The *p *values are annotated as follows: *p < 0.05; **p < 0.01; ***p < 0.005; **** *p* < 0.0001. For the analysis of the degradation of oxidized proteins by the four 20S proteasome subtypes, for the analysis of the degradation of p21 and c-myc by the four proteasome subtypes, for the analysis of the levels of hydrophobic patches present in native and oxidized calmodulin and hemoglobin, and for the analysis of the degradation of tau by the four 20S proteasome subtypes a one-way ANOVA followed by Bonferroni *post-hoc* comparison was performed. The *p-*values are annotated as follows: *p < 0.0332; **p < 0.0021; ***p < 0.0002; ****p < 0.0001.

## Supplementary information

Supplementary Information.

## References

[1] Dikic I (2017). Proteasomal and autophagic degradation systems. Annu. Rev. Biochem..

[2] Dengjel J (2005). Autophagy promotes MHC class II presentation of peptides from intracellular source proteins. Proc. Natl. Acad. Sci. USA.

[3] Rock KL (1994). Inhibitors of the proteasome block the degradation of most cell proteins and the generation of peptides presented on MHC class I molecules. Cell.

[4] Groll M (1997). Structure of 20S proteasome from yeast at 2.4 A resolution. Nature.

[5] Hisamatsu H (1996). Newly identified pair of proteasomal subunits regulated reciprocally by interferon gamma. J. Exp. Med..

[6] Guillaume B (2010). Two abundant proteasome subtypes that uniquely process some antigens presented by HLA class I molecules. Proc. Natl. Acad. Sci. USA.

[7] Vigneron N, Van den Eynde BJ (2014). Proteasome subtypes and regulators in the processing of antigenic peptides presented by class I molecules of the major histocompatibility complex. Biomolecules.

[8] de la Pena AH, Goodall EA, Gates SN, Lander GC, Martin A (2018). Substrate-engaged 26S proteasome structures reveal mechanisms for ATP-hydrolysis-driven translocation. Science (New York, N.Y.).

[9] Dong Y (2019). Cryo-EM structures and dynamics of substrate-engaged human 26S proteasome. Nature.

[10] Matyskiela ME, Lander GC, Martin A (2013). Conformational switching of the 26S proteasome enables substrate degradation. Nat. Struct. Mol. Biol..

[11] Unverdorben P (2014). Deep classification of a large cryo-EM dataset defines the conformational landscape of the 26S proteasome. Proc. Natl. Acad. Sci. USA.

[12] Huber EM (2012). Immuno- and constitutive proteasome crystal structures reveal differences in substrate and inhibitor specificity. Cell.

[13] Vigneron N, Abi Habib J, Van den Eynde BJ (2015). The capture proteasome assay: a method to measure proteasome activity in vitro. Anal. Biochem..

[14] Guillaume B (2012). Analysis of the processing of seven human tumor antigens by intermediate proteasomes. J. Immunol..

[15] Kincaid EZ (2012). Mice completely lacking immunoproteasomes show major changes in antigen presentation. Nat. Immunol..

[16] Van den Eynde BJ, Morel S (2001). Differential processing of class-I-restricted epitopes by the standard proteasome and the immunoproteasome. Curr. Opin. Immunol..

[17] Driscoll J, Brown MG, Finley D, Monaco JJ (1993). MHC-linked LMP gene products specifically alter peptidase activities of the proteasome. Nature.

[18] Gaczynska M, Rock KL, Goldberg AL (1993). Gamma-interferon and expression of MHC genes regulate peptide hydrolysis by proteasomes. Nature.

[19] Hayashi T, Faustman D (2000). Essential role of human leukocyte antigen-encoded proteasome subunits in NF-kappaB activation and prevention of tumor necrosis factor-alpha-induced apoptosis. J. Biol. Chem..

[20] Hensley SE (2010). Unexpected role for the immunoproteasome subunit LMP2 in antiviral humoral and innate immune responses. J. Immunol..

[21] Seifert U (2010). Immunoproteasomes preserve protein homeostasis upon interferon-induced oxidative stress. Cell.

[22] Bitzer A, Basler M, Krappmann D, Groettrup M (2017). Immunoproteasome subunit deficiency has no influence on the canonical pathway of NF-kappaB activation. Mol. Immunol..

[23] Nathan J (2013). Immuno- and constitutive proteasomes do not differ in their abilities to degrade ubiquitinated proteins. Cell.

[24] Runnels HA, Watkins WA, Monaco JJ (2000). LMP2 expression and proteasome activity in NOD mice. Nat. Med..

[25] Davies KJ (2001). Degradation of oxidized proteins by the 20S proteasome. Biochimie.

[26] Inai Y, Nishikimi M (2002). Increased degradation of oxidized proteins in yeast defective in 26 S proteasome assembly. Arch. Biochem. Biophys..

[27] Shringarpure R, Grune T, Mehlhase J, Davies KJ (2003). Ubiquitin conjugation is not required for the degradation of oxidized proteins by proteasome. J. Biol. Chem..

[28] De M (2003). Beta 2 subunit propeptides influence cooperative proteasome assembly. J. Biol. Chem..

[29] Griffin TA (1998). Immunoproteasome assembly: cooperative incorporation of interferon gamma (IFN-gamma)-inducible subunits. J. Exp. Med..

[30] Heink S, Ludwig D, Kloetzel PM, Kruger E (2005). IFN-gamma-induced immune adaptation of the proteasome system is an accelerated and transient response. Proc. Natl. Acad. Sci. USA.

[31] Schmidtke G, Emch S, Groettrup M, Holzhutter HG (2000). Evidence for the existence of a non-catalytic modifier site of peptide hydrolysis by the 20 S proteasome. J. Biol. Chem..

[32] Misra M (2017). Dissecting the specificity of adenosyl sulfamate inhibitors targeting the ubiquitin-activating enzyme. Structure (London, England: 1993).

[33] Hjerpe R (2009). Efficient protection and isolation of ubiquitylated proteins using tandem ubiquitin-binding entities. EMBO Rep..

[34] Amador V, Ge S, Santamaría PG, Guardavaccaro D, Pagano M (2007). APC/C(Cdc20) controls the ubiquitin-mediated degradation of p21 in prometaphase. Mol. Cell.

[35] Bornstein G (2003). Role of the SCFSkp2 ubiquitin ligase in the degradation of p21Cip1 in S phase. J. Biol. Chem..

[36] Farrell AS, Sears RC (2014). MYC degradation. Cold Spring Harbor Perspect. Med..

[37] Lee EW (2009). Differential regulation of p53 and p21 by MKRN1 E3 ligase controls cell cycle arrest and apoptosis. EMBO J..

[38] Lu Z, Hunter T (2010). Ubiquitylation and proteasomal degradation of the p21(Cip1), p27(Kip1) and p57(Kip2) CDK inhibitors. Cell Cycle (Georgetown, Tex.).

[39] Nishitani H (2008). CDK inhibitor p21 is degraded by a proliferating cell nuclear antigen-coupled Cul4-DDB1Cdt2 pathway during S phase and after UV irradiation. J. Biol. Chem..

[40] Reinheckel T, Ullrich O, Sitte N, Grune T (2000). Differential impairment of 20S and 26S proteasome activities in human hematopoietic K562 cells during oxidative stress. Arch. Biochem. Biophys..

[41] Wang X (2017). The proteasome-interacting Ecm29 protein disassembles the 26S proteasome in response to oxidative stress. J. Biol. Chem..

[42] Wang X, Yen J, Kaiser P, Huang L (2010). Regulation of the 26S proteasome complex during oxidative stress. Sci. Signal..

[43] Fabre B (2014). Comparison of label-free quantification methods for the determination of protein complexes subunits stoichiometry. EuProt.

[44] Fabre B (2013). Subcellular distribution and dynamics of active proteasome complexes unraveled by a workflow combining in vivo complex cross-linking and quantitative proteomics. Mol. Cell. Proteomics.

[45] Fabre B (2014). Label-free quantitative proteomics reveals the dynamics of proteasome complexes composition and stoichiometry in a wide range of human cell lines. J. Proteome Res..

[46] Smith DM (2007). Docking of the proteasomal ATPases' carboxyl termini in the 20S proteasome's alpha ring opens the gate for substrate entry. Mol. Cell.

[47] Wallace WJ, Houtchens RA, Maxwell JC, Caughey WS (1982). Mechanism of autooxidation for hemoglobins and myoglobins. Promotion of superoxide production by protons and anions. J. Biol. Chem..

[48] Driscoll J, Goldberg AL (1989). Skeletal muscle proteasome can degrade proteins in an ATP-dependent process that does not require ubiquitin. Proc. Natl. Acad. Sci. USA.

[49] Kisselev AF, Akopian TN, Goldberg AL (1998). Range of sizes of peptide products generated during degradation of different proteins by archaeal proteasomes. J. Biol. Chem..

[50] Raynes R, Pomatto LC, Davies KJ (2016). Degradation of oxidized proteins by the proteasome: Distinguishing between the 20S, 26S, and immunoproteasome proteolytic pathways. Mol. Asp. Med..

[51] Ferrington DA (2001). Selective degradation of oxidized calmodulin by the 20 S proteasome. J. Biol. Chem..

[52] Sreerama N, Woody RW (2000). Estimation of protein secondary structure from circular dichroism spectra: comparison of CONTIN, SELCON, and CDSSTR methods with an expanded reference set. Anal. Biochem..

[53] Whitmore L, Wallace BA (2008). Protein secondary structure analyses from circular dichroism spectroscopy: methods and reference databases. Biopolymers.

[54] Eikawa S (2013). Induction of CD8 T-cell responses restricted to multiple HLA class I alleles in a cancer patient by immunization with a 20-mer NY-ESO-1f (NY-ESO-1 91-110) peptide. Int. J. Cancer.

[55] Sourdive DJD (1998). Conserved T Cell Receptor Repertoire in Primary and Memory CD8 T Cell Responses to an Acute Viral Infection. J. Exp. Med..

[56] Muchamuel T (2009). A selective inhibitor of the immunoproteasome subunit LMP7 blocks cytokine production and attenuates progression of experimental arthritis. Nat. Med..

[57] Asher G, Bercovich Z, Tsvetkov P, Shaul Y, Kahana C (2005). 20S proteasomal degradation of ornithine decarboxylase is regulated by NQO1. Mol. Cell.

[58] David DC (2002). Proteasomal degradation of tau protein. J. Neurochem..

[59] Grune T (2010). Tau protein degradation is catalyzed by the ATP/ubiquitin-independent 20S proteasome under normal cell conditions. Arch. Biochem. Biophys..

[60] Menneteau T (2019). Mass spectrometry-based absolute quantification of 20S proteasome status for controlled expansion of human adipose-derived mesenchymal stromal/stem cells. Mol. Cell. Proteomics.

[61] Bard JAM, Bashore C, Dong KC, Martin A (2019). The 26S proteasome utilizes a kinetic gateway to prioritize substrate degradation. Cell.

[62] Benaroudj N, Zwickl P, Seemuller E, Baumeister W, Goldberg AL (2003). ATP hydrolysis by the proteasome regulatory complex PAN serves multiple functions in protein degradation. Mol. Cell.

[63] Henderson A, Erales J, Hoyt MA, Coffino P (2011). Dependence of proteasome processing rate on substrate unfolding. J. Biol. Chem..

[64] Fabre B (2015). Deciphering preferential interactions within supramolecular protein complexes: the proteasome case. Mol. Syst. Biol..

[65] Lee BH (2010). Enhancement of proteasome activity by a small-molecule inhibitor of USP14. Nature.

[66] Manohar S (2019). Polyubiquitin chains linked by lysine residue 48 (K48) selectively target oxidized proteins in vivo. Antioxid. Redox Signal..

[67] Medicherla B, Goldberg AL (2008). Heat shock and oxygen radicals stimulate ubiquitin-dependent degradation mainly of newly synthesized proteins. J. Cell Biol..

[68] Grune T, Reinheckel T, Davies KJ (1996). Degradation of oxidized proteins in K562 human hematopoietic cells by proteasome. J. Biol. Chem..

[69] Huang Q, Wang H, Perry SW, Figueiredo-Pereira ME (2013). Negative regulation of 26S proteasome stability via calpain-mediated cleavage of Rpn10 subunit upon mitochondrial dysfunction in neurons. J. Biol. Chem..

[70] Iwai K (2012). Diverse ubiquitin signaling in NF-kappaB activation. Trends Cell Biol..

[71] Armstrong JA (2018). Oxidative stress alters mitochondrial bioenergetics and modifies pancreatic cell death independently of cyclophilin D, resulting in an apoptosis-to-necrosis shift. J. Biol. Chem..

[72] Green K, Brand MD, Murphy MP (2004). Prevention of mitochondrial oxidative damage as a therapeutic strategy in diabetes. Diabetes.

[73] Sabharwal SS, Schumacker PT (2014). Mitochondrial ROS in cancer: initiators, amplifiers or an Achilles' heel?. Nat. Rev. Cancer.

[74] Groll M (2000). A gated channel into the proteasome core particle. Nat. Struct. Biol..

[75] Kisselev AF, Kaganovich D, Goldberg AL (2002). Binding of hydrophobic peptides to several non-catalytic sites promotes peptide hydrolysis by all active sites of 20 S proteasomes. Evidence for peptide-induced channel opening in the alpha-rings. J. Biol. Chem..

[76] Kisselev AF, Callard A, Goldberg AL (2006). Importance of the different proteolytic sites of the proteasome and the efficacy of inhibitors varies with the protein substrate. J. Biol. Chem..

[77] Li J, Powell SR, Wang X (2011). Enhancement of proteasome function by PA28α overexpression protects against oxidative stress. FASEB J..

[78] Pickering AM (2010). The immunoproteasome, the 20S proteasome, and the PA28αβ proteasome regulator are oxidative-stress-adaptative proteolytic complexes. Biochem. J..

[79] Pickering AM, Linder RA, Zhang H, Forman HJ, Davies KJA (2012). Nrf2-dependent induction of proteasome and Pa28αβ regulator are required for adaptation to oxidative stress. J. Biol. Chem..

[80] Schultz ES (2002). The production of a new MAGE-3 peptide presented to cytolytic T lymphocytes by HLA-B40 requires the immunoproteasome. J. Exp. Med..

[81] Jentoft N, Dearborn DG (1979). Labeling of proteins by reductive methylation using sodium cyanoborohydride. J. Biol. Chem..

[82] Besche HC, Haas W, Gygi SP, Goldberg AL (2009). Isolation of mammalian 26S proteasomes and p97/VCP complexes using the ubiquitin-like domain from HHR23B reveals novel proteasome-associated proteins. Biochemistry.

[83] Ando K (2020). Picalm reduction exacerbates tau pathology in a murine tauopathy model. Acta Neuropathol..

[84] Brion JP (1991). Tau in Alzheimer neurofibrillary tangles. N- and C-terminal regions are differentially associated with paired helical filaments and the location of a putative abnormal phosphorylation site. Biochem. J..

