# Supplementary material for: Efficiency of the four proteasome subtypes to degrade ubiquitinated or oxidized proteins
Source: Sci Rep. 2020 Sep 25;10:15765. doi: 10.1038/s41598-020-71550-5 (PMC7519072; doi:10.1038/s41598-020-71550-5)
Supplement: Supplementary file 1 — Supplementary Information. [file 41598_2020_71550_MOESM1_ESM.pdf]

Efficiency of the four proteasome subtypes to degrade ubiquitinated or oxidized proteins

Joanna Abi Habib[1,2,3], Etienne De Plaen[1,2,3], Vincent Stroobant[1,2,3], Dusan Zivkovic[4], Marie-Pierre Bousquet[4], Benoît Guillaume[1,2,5], Khadija Wahni[6,7,8], Joris Messens[6,7,8], Antonia Busse[9,10], Nathalie Vigneron[1,2,3,11*], Benoit J. Van den Eynde[1,2,3,11*]

(1) Ludwig Institute for Cancer Research, Brussels, Belgium
(2) de Duve Institute, UCLouvain, Brussels, Belgium
(3) WELBIO (Walloon Excellence in Life Sciences and Biotechnology), Brussels, Belgium.
(4) Institut de Pharmacologie et Biologie Structurale, IPBS, Université de Toulouse, CNRS, UPS, Toulouse, France
(5) Centre hospitalier de Jolimont, Service de biochimie médicale, La Louvière, Belgium.
(6) Structural Biology Research Center, VIB, 1050 Brussels, Belgium
(7) Structural Biology Brussels Laboratory, Vrije Universiteit Brussel, 1050 Brussels, Belgium
(8) Brussels Center for Redox Biology, 1050 Brussels, Belgium.
(9) Medizinische Klinik III, Hämatologie, Onkologie und Tumorimmunologie Charité – Universitätsmedizin Berlin, corporate member of Freie Universität Berlin, Humboldt-Universität zu Berlin, and Berlin Institute of Health, Campus Benjamin Franklin, Hindenburgdamm 30, 12203 Berlin
(10) German Cancer Consortium (DKTK), partner site Berlin and German Cancer Research Center (DKFZ), Heidelberg, Germany
(11) These authors contributed equally to this work
* Correspondence : nathalie.vigneron@bru.licr.org (NVI)
benoit.vandeneynde@bru.licr.org (BVDE) (Lead contact)

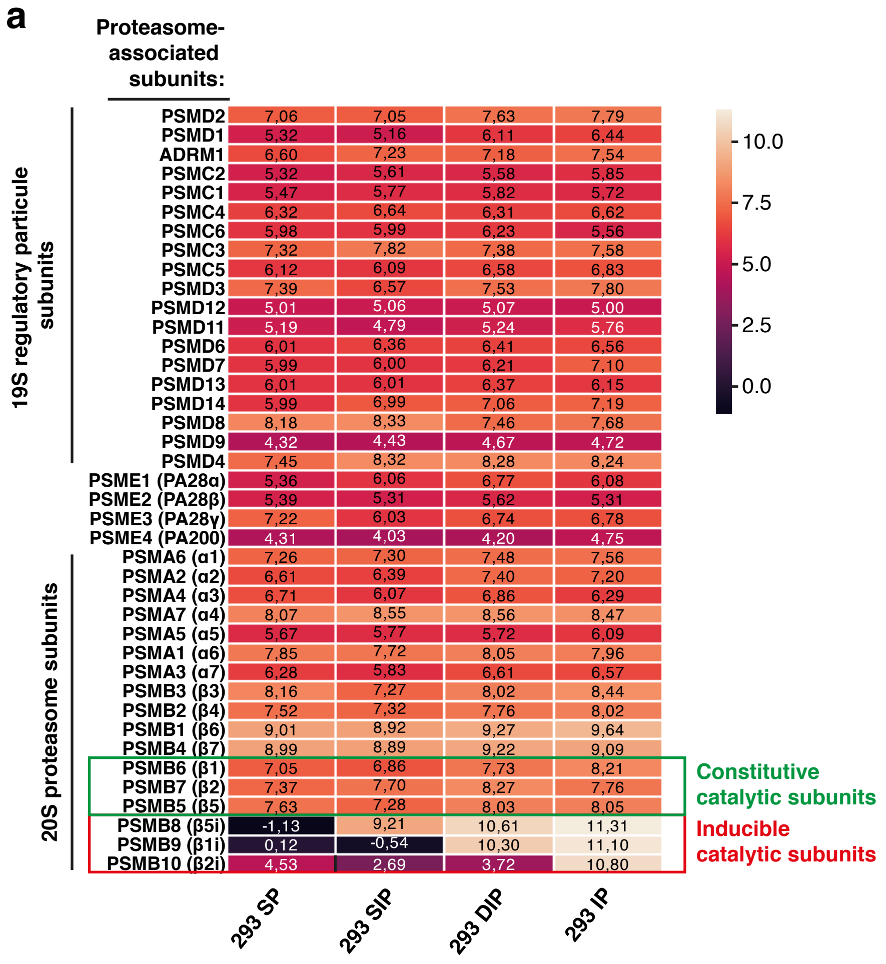

Supplementary Figure 1. Characterization of the proteasome content of the four 293 cell lines that were used. (**a**) RNAseq analysis performed on the four 293 cell lines: heatmap of the log2 of the TPM (transcripts per million mapped reads) counts of proteasome subunit genes. (**b**) Immunodetection of the constitutive and inducible catalytic subunits in the total lysates of the four 293 cell lines. For PSMB8 (β5i), PSMB9 (β1i) and PSMB10 (β2i), the lower bands correspond to the mature active subunits, whereas the upper bands correspond to the immature subunits containing the N-terminal propeptides. The primary antibodies that were used: mouse anti-β1 monoclonal antibody from enzo life science BML-PW8140; mouse anti-β2 monoclonal antibody from abcam ab22650; rabbit anti-β5 polyclonal antibody from abcam ab3330; mouse anti-β1i monoclonal antibody CH3-scB2; mouse anti-β2i monoclonal antibody 9E9cl3 and polyclonal rabbit anti-β5i from abcam ab3329 and mouse anti-vinculin monoclonal antibody from Sigma V9131. The CH3-scB2 and 9E9cl3 antibodies, recognizing respectively the β1i and β2i subunits of proteasome were purified from the supernatant of the respective hybridomas, which were obtained following B6 mice immunization with either the CLPKFYDE (β1i) or CAMEVE (β2i) peptides conjugated to maleimide-activated ovalbumin.

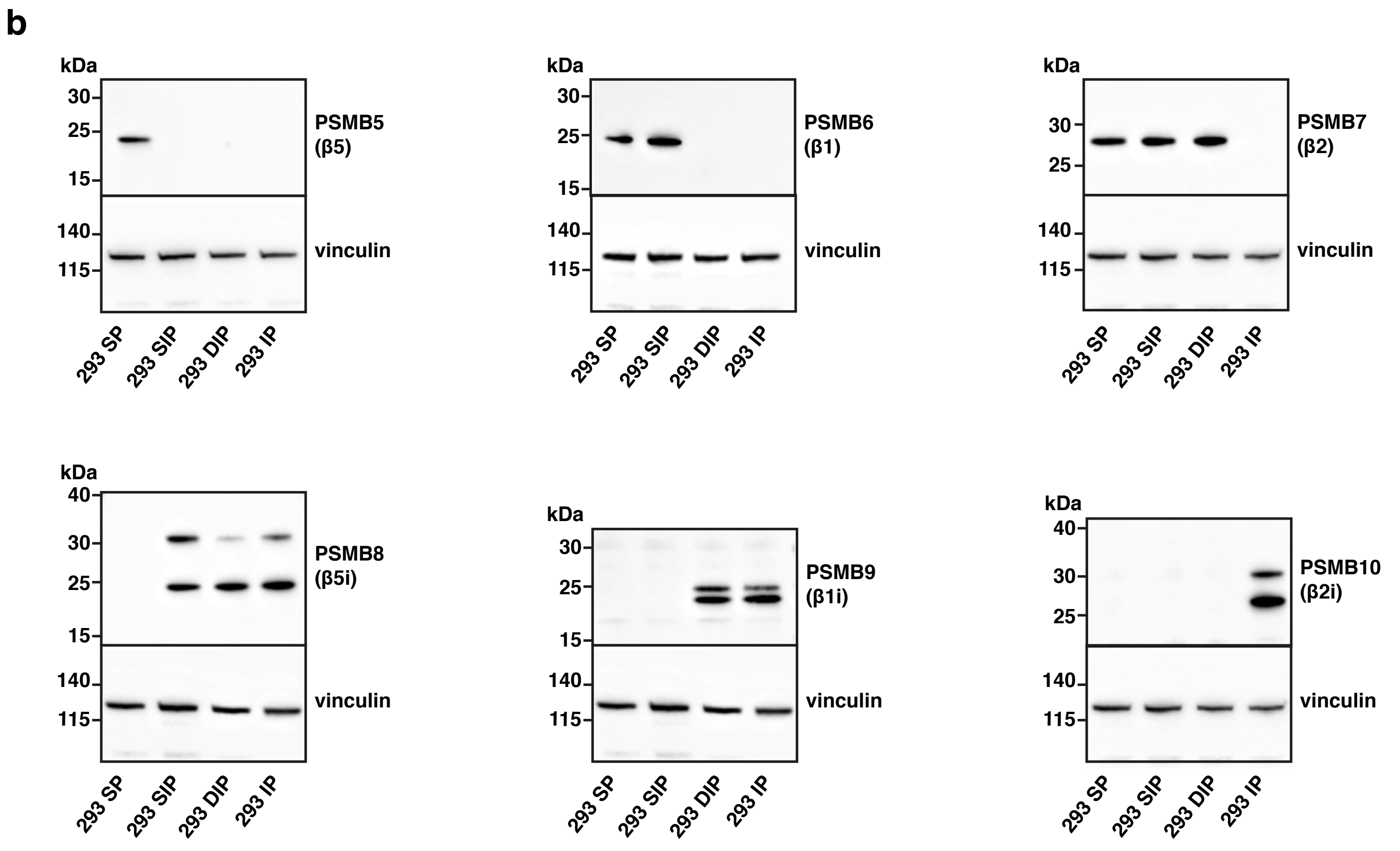

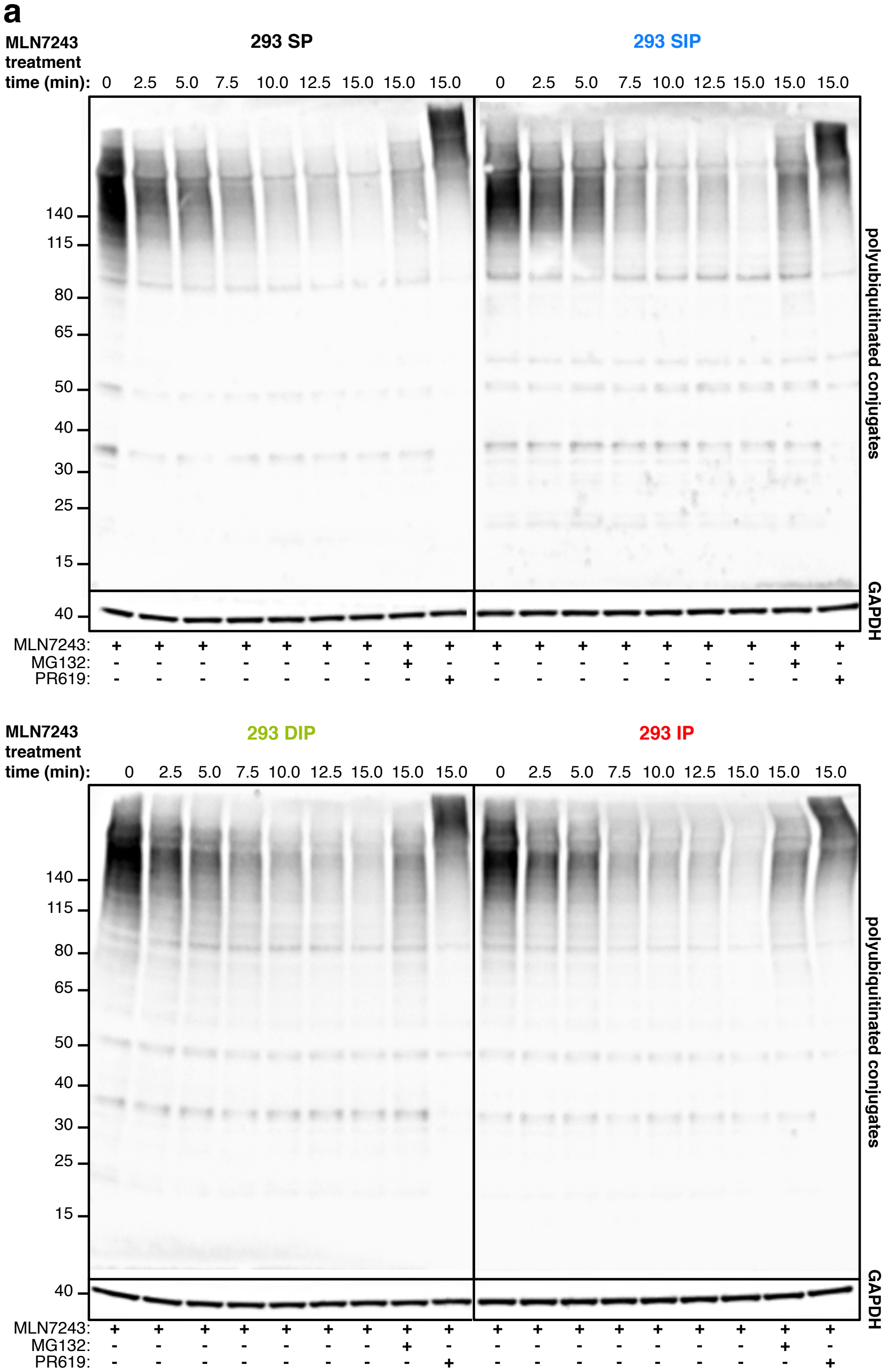

Supplementary Figure 2. Kinetics of the clearance of polyubiquitinated proteins in the four 293 cell lines treated with 5 µM MLN7243, an inhibitor of the E1 ubiquitin-activating enzyme.
(**a**) Western blot analysis of lysates of cells treated with 5 µM of MLN7243 and collected every 2.5 minutes and this for 15 minutes. To monitor the role of proteasome and deubiquitinases in the clearance of polyubiquitinated proteins, cells were treated with 20 µM of MG132 (Selleck Chemicals) and 5 µM of PR619 (Selleck Chemicals), a non selective inhibitor of deubiquitinases, prior to MLN7243 treatment (2 right lanes of the four Western blots). Polyubiquitinated proteins were detected using the FK1 antibody (BML-PW8805-0500) and GAPDH was detected using GAPDH antibody (Cat# 2118). (**b**) Densitometric evaluation of the kinetics of clearance of polyubiquitinated proteins in the four 293 cell lines treated with 5 µM MLN7243. All values (+SEM) are collected from three independent experiments. For the analysis of the clearance of polyubiquitinated proteins in the four 293 cells lines treated with MLN7243, a one-way ANOVA followed by Bonferroni post-hoc comparison was performed. (**c**) Densitometric evaluation of the levels of polyubiquitinated proteins after 15 minutes of treatment with 5 µM of MLN7243 in the presence or absence of MG132. All values (+SEM) are collected from two independent experiments. There is no statistically significant difference between the levels of polyubiquitinated proteins between the four cell lines treated with 20 µM of MG132.

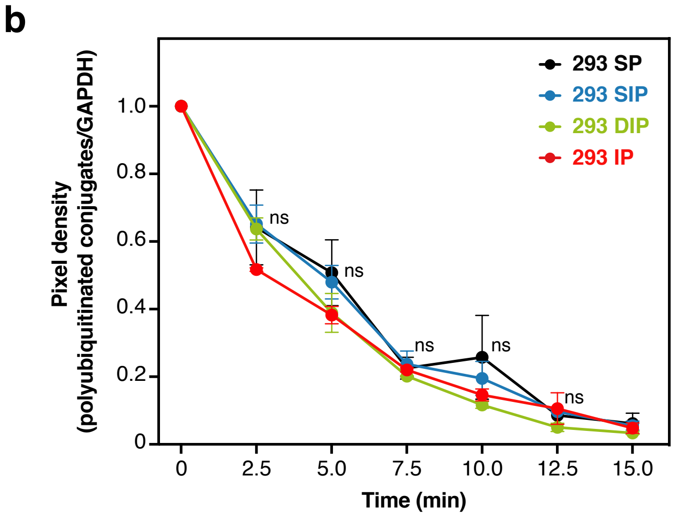

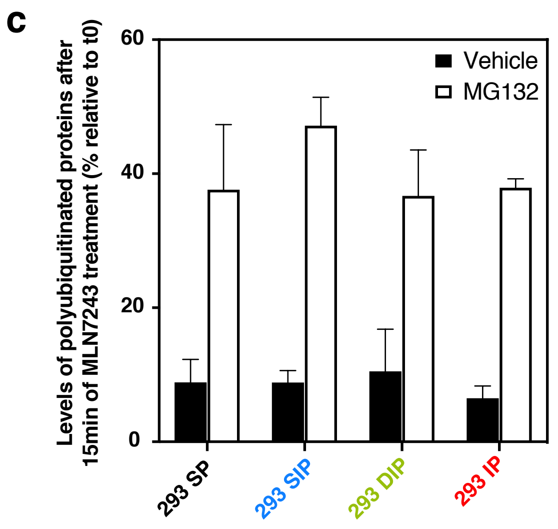

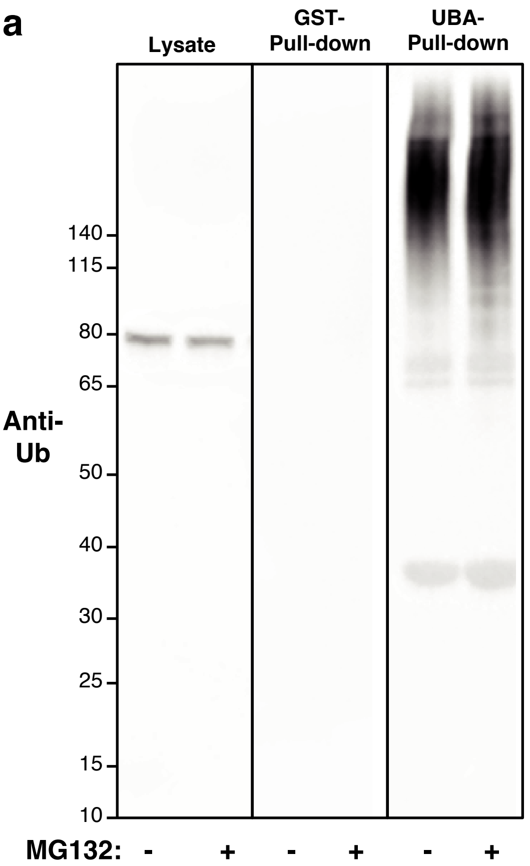

Supplementary Figure 3. p21 and c-myc are ubiquitinated.

Isolation of the endogenously ubiquitinated proteins using the tandem ubiquitin binding entity (TUBE)-pull down assay (Hjerpe et al., 2009). 293 cells were treated or not, for 4 h, with 20 µM of the proteasome inhibitor MG132 and lysed in lysis buffer (20 mM sodium phosphate pH 7.4, 1% NP-40, 2 mM EDTA, 1 mM DTT, 50 mM N-Ethylmaleimide). The lysate was then incubated with glutathione beads, which were pretreated with either 30 µg of GST as control or with 30 µg of GST-UBA fusion protein. After an overnight incubation, the glutathione beads were pulled-down and proteins were eluted by heating the beads for 25 min in 40 µL of 1.5x NU-PAGE LDS sample buffer. Lysates, GST pull-down and GST-UBA pull-down were then analyzed by immunoblotting using either (**a**) the FK1 anti-ubiquitin antibody to evaluate the efficiency of the TUBE-pull down assay to isolate ubiquitinated proteins, (**b**) a p21 antibody or (**c**) a c-myc antibody to evaluate the presence of polyubiquitinated p21 and c-myc respectively.

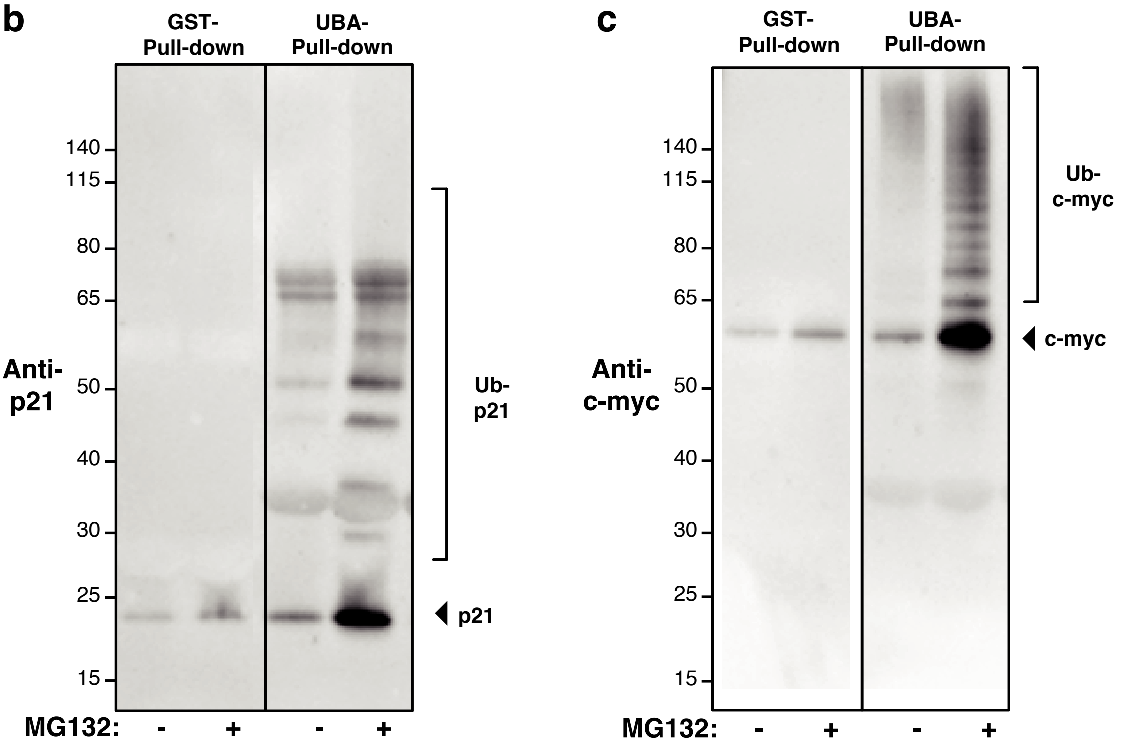

Genes associated with ubiquitination:

Group	Gene	293 SP	293 SIP	293 DIP	293 IP
Ubiquitin-activating enzymes (E1)	UBA1	9,19	8,77	8,96	8,39
	UBA6	2,43	2,96	3,13	3,06
Ubiquitin-conjugating enzymes (E2)	BIRC6	3,14	3,34	3,39	3,96
	UBE2A	4,92	5,03	5,48	5,70
	UBE2B	3,05	3,01	3,50	3,59
	UBE2C	7,34	8,29	7,82	8,26
	UBE2D1	2,62	2,60	2,72	2,99
	UBE2D2	4,92	4,84	5,43	4,89
	UBE2D3	5,90	6,58	6,26	6,71
	UBE2D4	3,69	2,83	3,12	2,71
	UBE2E1	5,98	6,54	5,42	7,01
	UBE2E2	5,27	5,65	4,89	5,43
	UBE2G1	3,41	4,05	4,27	4,54
	UBE2G2	4,69	4,10	4,78	4,75
	UBE2H	4,83	4,82	4,15	4,23
	UBE2J1	4,30	4,14	4,33	4,41
	UBE2J2	5,06	5,18	5,58	5,84
	UBE2K	4,81	5,15	4,36	4,83
	UBE2L3	6,08	5,80	6,14	5,90
	UBE2O	5,39	6,25	5,60	5,79
	UBE2Q1	5,70	5,15	5,64	5,50
	UBE2Q2	3,97	3,41	3,16	2,82
	UBE2R1	6,79	6,69	6,47	6,71
	UBE2R2	5,00	5,33	4,94	5,26
	UBE2S	8,05	7,69	7,55	7,64
	UBE2T	6,16	6,41	6,24	6,85
	UBE2W	2,65	2,53	2,22	2,10
	UBE2Z	5,56	5,69	5,79	5,82
Proteins associated with the ubiquitin ligases (E3) specific for p21 and c-myc	ANAPC1	4,67	4,63	4,68	4,91
	ANAPC2	4,20	4,38	4,58	4,86
	ANAPC4	3,72	4,04	3,70	3,29
	ANAPC5	6,40	6,91	7,17	6,58
	ANAPC7	6,03	6,32	5,87	6,07
	ANAPC10	3,52	3,54	3,76	3,36
	ANAPC11	7,30	6,33	7,16	6,72
	ANAPC13	6,04	5,48	6,47	5,86
	ANAPC15	4,78	4,21	4,94	5,62
	ANAPC16	5,04	4,50	4,88	4,31
	BTRC	3,24	3,02	3,80	4,11
	CDC16	4,60	4,59	5,04	4,61
	CDC20	7,86	8,06	8,32	8,63
	CDC23	5,15	5,29	5,78	6,29
	CDC26	5,63	5,02	5,97	5,38
	CDC27	5,08	4,85	5,61	5,92
	CKS1B	6,30	6,27	6,45	6,58
	CUL1	5,60	6,14	5,61	5,88
	CUL4A	5,40	4,38	5,02	5,34
	DDB1	7,99	7,94	8,05	8,38
	DTL	4,91	5,49	5,14	5,10
	FBXW7	1,99	2,25	2,03	2,29
	FBXW8	3,38	4,12	3,31	3,46
	HUWE1	6,36	6,60	6,54	6,19
	MKRN1	5,67	5,15	5,35	4,94
	RBX1	6,21	5,53	6,03	6,25
	SKP1	6,50	4,34	5,75	7,29
	SKP2	5,61	5,23	5,51	5,99
	STUB1	7,58	7,92	7,44	7,99
	TRIM32	3,96	4,90	4,68	4,21
	TRPC4AP	5,28	5,93	6,18	6,06
	USP39	6,73	6,24	6,17	6,75

Color scale: 0.0 – 2.0 – 4.0 – 6.0 – 8.0 – 10.0

Supplementary Figure 4. RNAseq analysis performed on the four 293 cell lines: heatmap of the log2 of the TPM (transcripts per million mapped reads) counts of genes of the E1 and the E2 enzymes that are associated with the ubiquitin proteasome system, and of the subunits of the E3 ubiquitin ligases responsible for the ubiquitination of p21 and c-myc.

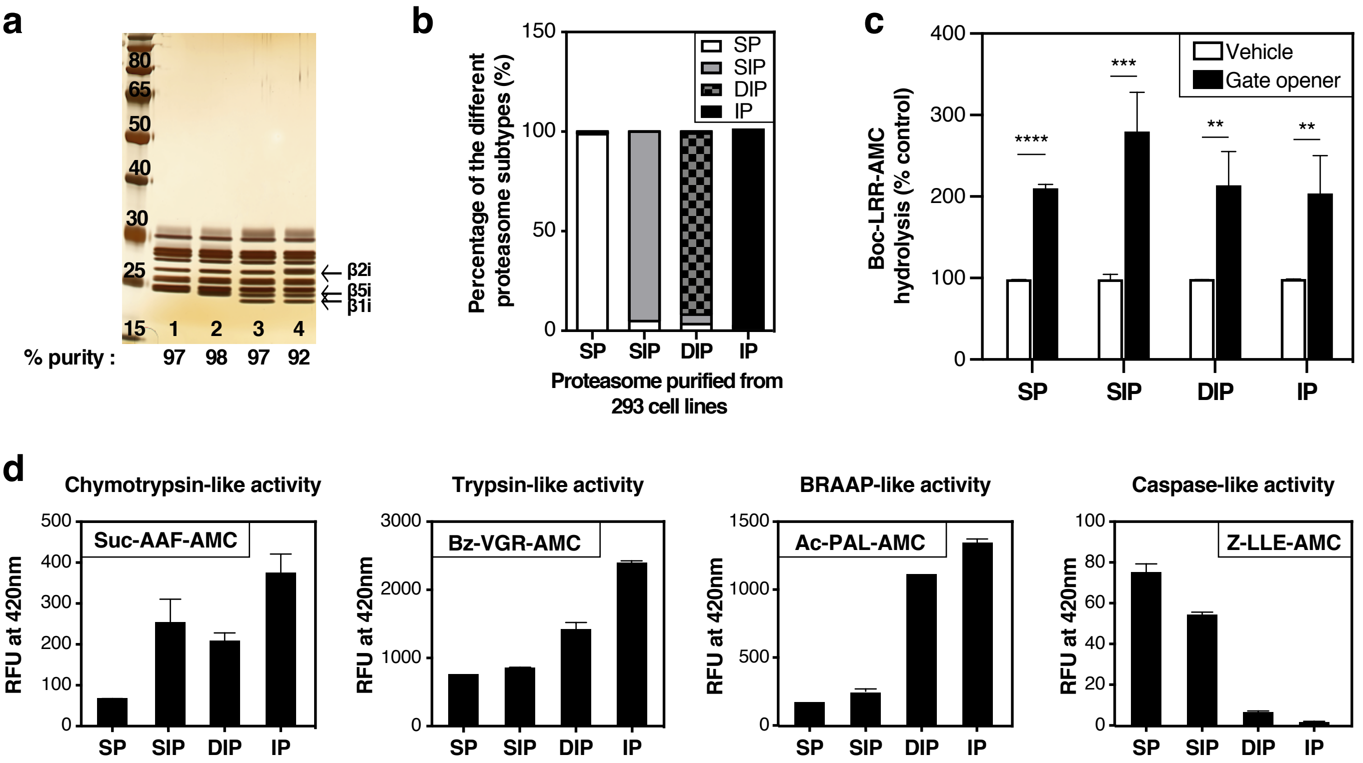

Supplementary Figure 5. Validation of the purified proteasome preparations. (**a**) Purity of the SP (lane 1), the SIP (lane 2), the DIP (lane 3) and the IP (lane 4) preparations as evaluated by silver staining of a polyacrylamide gel loaded with each proteasome preparation. The different bands correspond to the 14 distinct subunits of the 20S proteasomes. Given that these 14 subunits have similar molecular weight some of them overlap in this one dimensional polyacrylamide gel. The percentages shown below correspond to the purity of the proteasome preparations as evaluated by LC-MS/MS. (**b**) Validation by LC-MS/MS proteomic analysis of the presence of only one proteasome subtype in each preparation of purified proteasome from the four 293 cell lines. (**c**) Examining the conformation of the gates of the four proteasome subtypes. Proteasomes were treated or not with the 8-amino acid peptide GTPEGLYL derived from the Rpt2 subunit of the 19S RP and referred to as "gate opener". To monitor gate opening, hydrolysis of the fluorogenic substrate Boc-LRR-AMC when proteasomes were treated with the "gate opener" was compared to the control condition (Vehicle). These values are means of duplicates from two independent experiment +SD (**$p<0.005$, ***$p<0.0005$ ****$p<0.0001$, unpaired t-test). (**d**) Analysis of the different activities of the four proteasome subtypes by evaluating their ability to degrade 100µM of different fluorogenic substrates that are specific for each activity and that fluoresce at 420nm when degraded. These values are means of duplicates from one experiment +SD.

Supplementary Figure 6. 26S proteasomes are unable to degrade oxidized calmodulin in an ubiquitin-independent manner.

(**a**) Western blot analysis on the purified 20S double intermediate proteasome (lane1) and the four purified 26S proteasomes, 26S SP (lane2), 26S SIP (lane3), 26S DIP (lane4) and 26S IP (lane5). The primary antibodies that were used: for the upper blot, rabbit polyclonal anti-proteasome 19S S4 antibody (ab140450) and for the lower blot, rabbit polyclonal anti-proteasome 20S core subunits antibody (BML-PW8155). The latter was obtained by immunizing rabbits with native 20S complexes, and therefore recognizes several subunits of the 20S complex. (**b**) Analysis of the different activities of the four 20S and 26S proteasome subtypes by evaluating their ability to degrade, in the presence of 100 µM of ATP, 10µM of different fluorogenic substrates that are specific for each activity and that fluoresce at 420nm when degraded. These values are means of duplicates from one experiment +SD. (**c**) Western blot analysis of the kinetics of degradation of oxidized calmodulin by the four 26S proteasome subtypes and by the 20S double intermediate proteasome. 1.5µM of oxidized calmodulin were mixed with 0.025µM of purified 26S proteasome or 20S proteasome in a buffer containing 5 mM Tris-HCl pH 7.4, 1 mM $MgCl_2$, 10 mM KCl, 0.01 mM EGTA and 2mM of ATP. Similar results were obtained from three independent experiments.

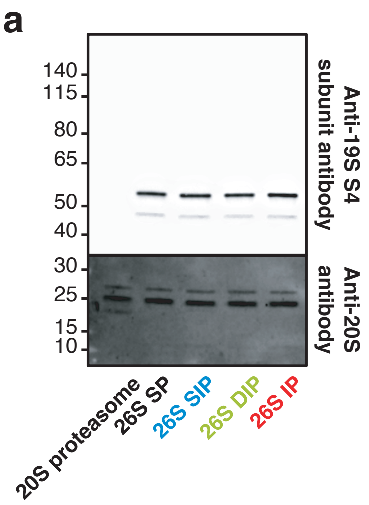

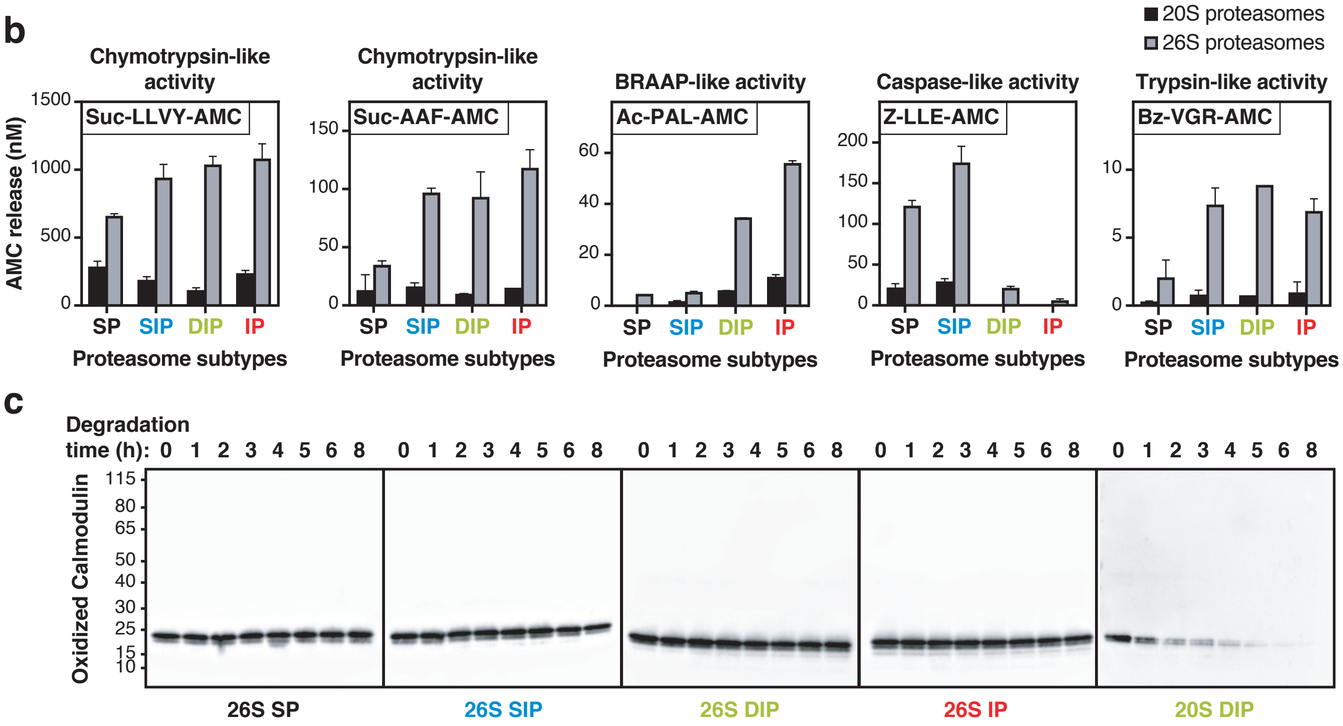

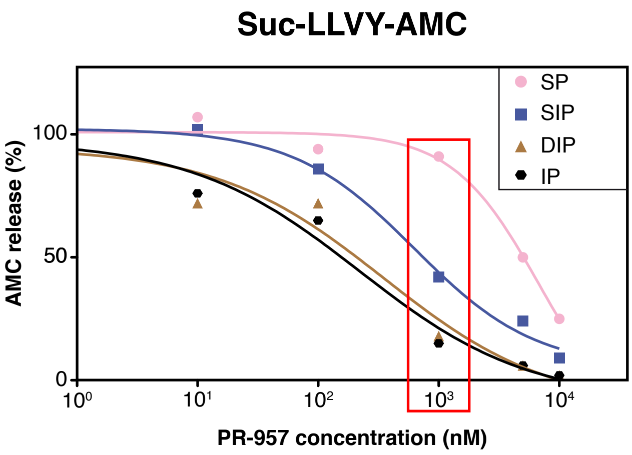

Supplementary Figure 7. Inhibition of the activity of the β5i subunit with PR-957. Dose curves assessing the concentration of PR-957 to be used to specifically block the chymo-trypsin-like activity linked to the catalytic subunit β5i. Increasing concentrations of the PR-957 inhibitor were added to the different proteasomes. Inhibition of the chymotrypsin-like activity of proteasomes was analyzed by monitoring the release of the fluorescent AMC upon degradation of the fluorogenic substrate Suc-LLVY-AMC.

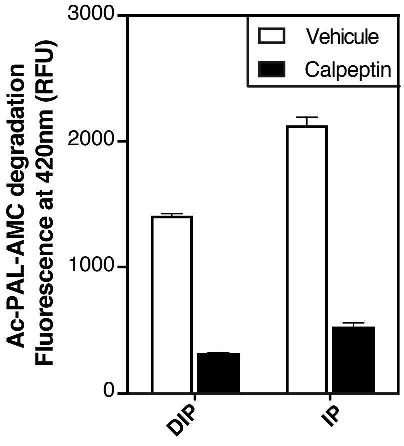

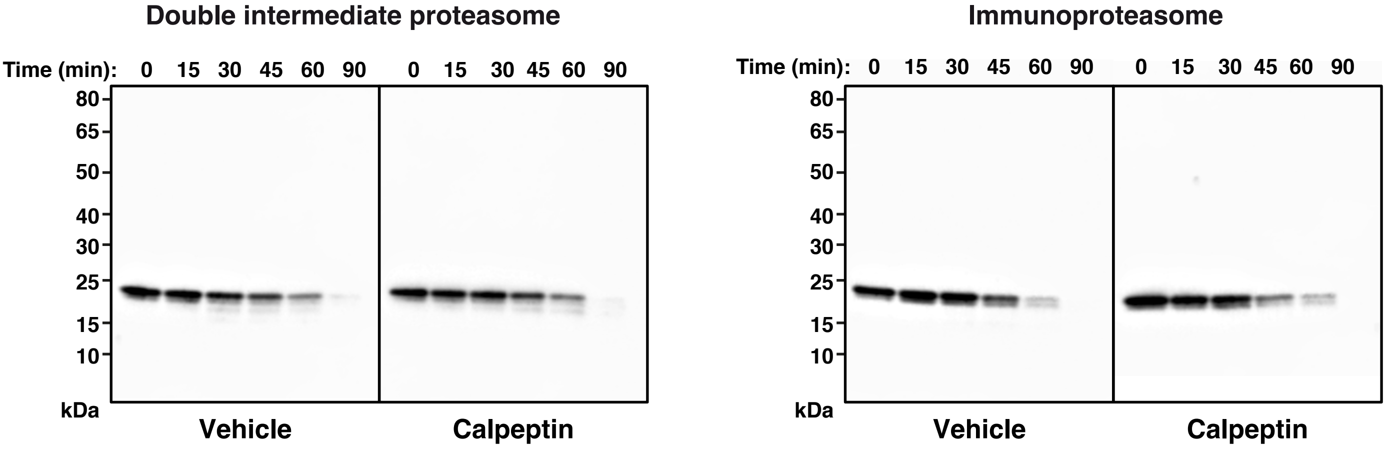

Supplementary Figure 8. Subunit β1i does not play a role in the degradation of oxidized proteins.
(**a**) Calpeptin blocks the β1i catalytic subunit of the proteasome. IP and DIP were incubated with 1.5 µM of Calpeptin prior to the addition of the β1i-specific fluorogenic substrate Ac-PAL-AMC. (**b**) Calpeptin does not inhibit the degradation of oxidized calmodulin by the DIP and the IP. The lack of effect on the degradation of oxidized calmodulin when DIP and IP are treated with calpeptin was shown in five independent experiments.

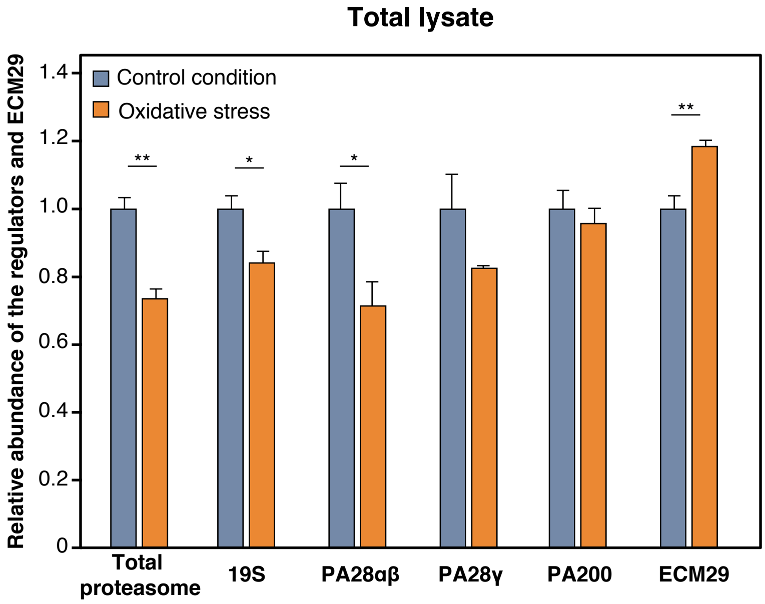

Supplementary Figure 9. The different proteasome regulators and ECM29 in lysates of cells treated or not with H_2O_2

Graph showing the ratio of the different proteasome regulators and ECM29 in total lysate obtained after cross-linking from cells treated with 2 mM of H_2O_2 (oxidized condition) as compared to the normal condition. The abundance of the indicated proteins was measured in control and oxidized conditions, normalized to the abundance of the 20S, and then the oxidized condition was normalized to the control condition. All values are means of three independent experiments ± SD. (* $p<0.05$ ** $p<0.01$ *** $p<0.001$, Student t-test).

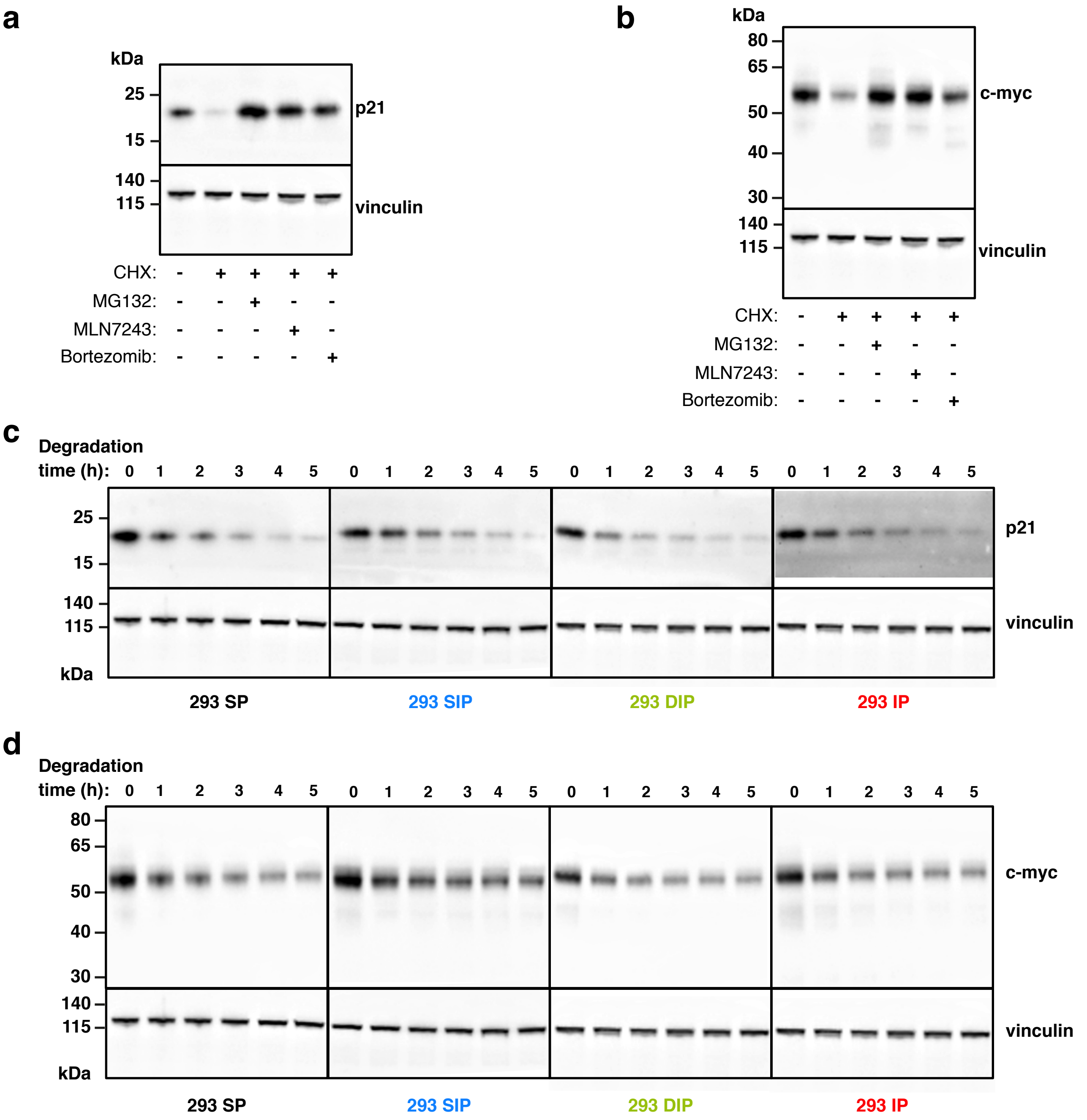

Supplementary Figure 10. Full-length images for the western blots included in Figure 1. Degradation of (**a**) p21 and (**b**) c-myc in a proteasome and ubiquitin -dependent manner. (**c**,**d**) Western blot analysis of the kinetics of degradation of (**c**) p21 and (**d**) c-myc in 293 cell lines expressing the different proteasome subtypes. To avoid stripping, the blots were cut in three parts. The lower parts from 15 to 25kDa were labelled with anti-p21 antibody (Cat#2947S), middle parts from 30 to 80kDa were labelled with anti-c-myc antibody (Cat#13987), and upper parts were labeled with anti-vinculin antibody (Cat#V9131).

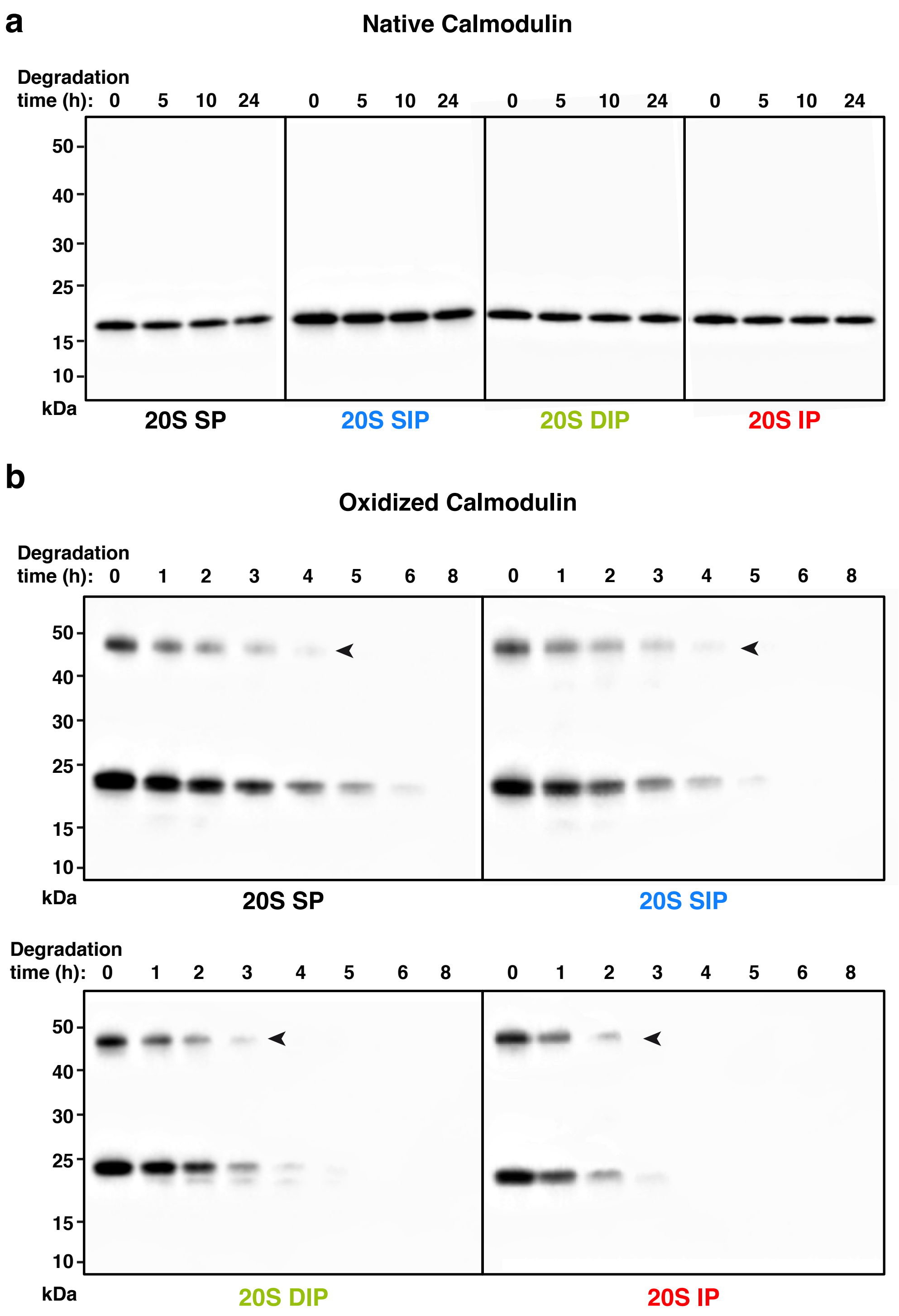

Supplementary Figure 11. Full-length images for the western blots included in Figure 3. Western blot analysis of the kinetics of degradation of (**a**) native and (**b**) oxidized calmodulin by the four 20S proteasome subtypes. (**a**) The native calmodulin has a molecular weight of 16.7kDa, and migrates to the expected position on the denaturing polyacrylamide gel. (**b**) The oxidized form of calmodulin has a molecular weight of 16.844kDa. The 144Da increase in the molecular weight is due to the oxidation of 9 methionines into methionine sulfoxide. As a result of its oxidation, calmodulin migrates slightly slower than expected. The reduced mobility of oxidized calmodulin was previously shown in the literature (Ferrington D.A. et al. JBC, 2001). The upper bands indicated by arrowheads are aggregates formed by two oxidized calmodulins that were not dissociated by the denaturing polyacrylamide gel. These oxidized calmodulin aggregates were not always observed in our experiments. Degradation of these oxidized calmodulin aggregates (upper bands) is similar to the degradation of the oxidized calmodulin (lower bands).

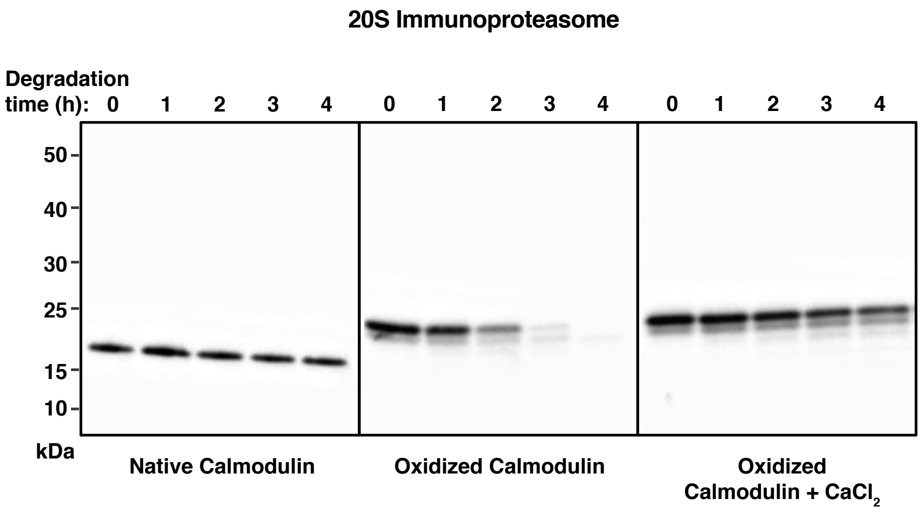

Supplementary Figure 12. Full-length images for the western blots included in Figure 4b. Western blot analysis of the kinetics of degradation of native calmodulin, oxidized calmodulin and oxidized calmodulin treated with 0.5 mM $CaCl_2$ by the immunoproteasome.

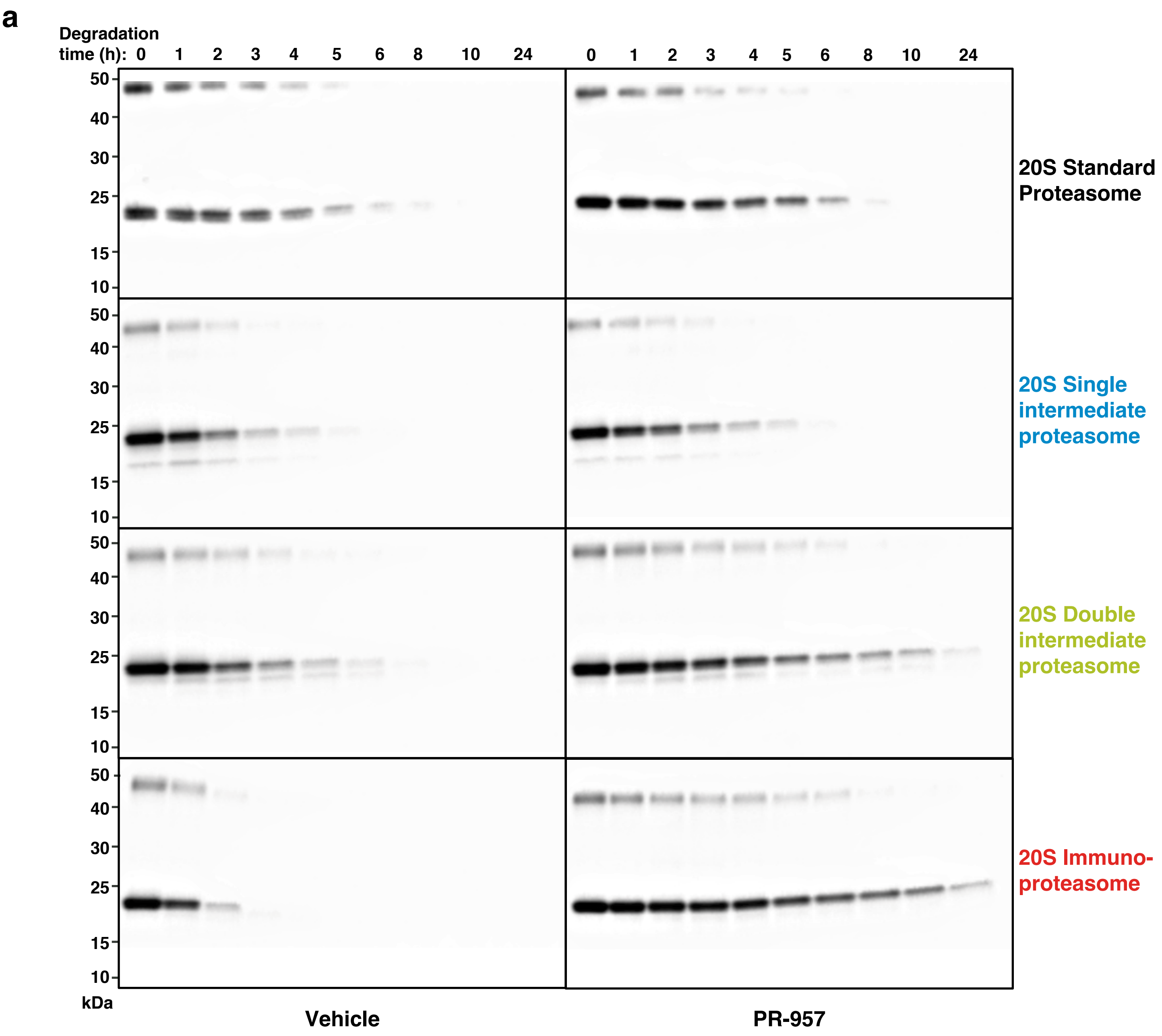

Supplementary Figure 13a. Full-length images for the western blots included in Figure 6a. Western blot analysis of the kinetics of degradation of oxidized calmodulin by the four proteasome subtypes in the presence or absence of the β5i-specific inhibitor PR-957. The upper bands are aggregates formed by two oxidized calmodulins that were not dissociated by the denaturing polyacrylamide gel. Degradation of these oxidized calmodulin aggregates (upper bands) is similar to the degradation of the oxidized calmodulin (lower bands).

b

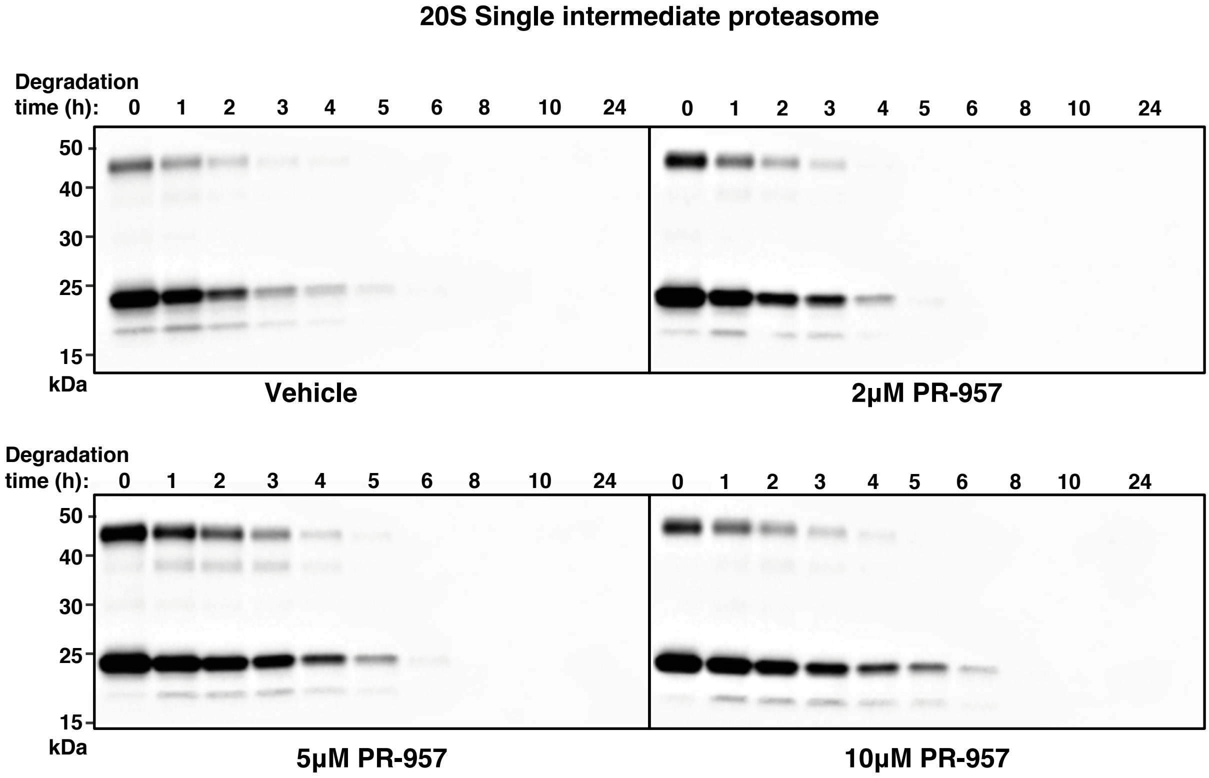

Supplementary Figure 13b. Full-length images for the western blots included in Figure 6c. Western blot analysis of the kinetics of degradation of oxidized calmodulin by the intermediate proteasome β5i treated with increased concentrations of the PR-957 inhibitor**.** The upper bands are aggregates formed by two oxidized calmodulins that were not dissociated by the denaturing polyacrylamide gel. Degradation of these oxidized calmodulin aggregates (upper bands) is similar to the degradation of the oxidized calmodulin (lower bands).

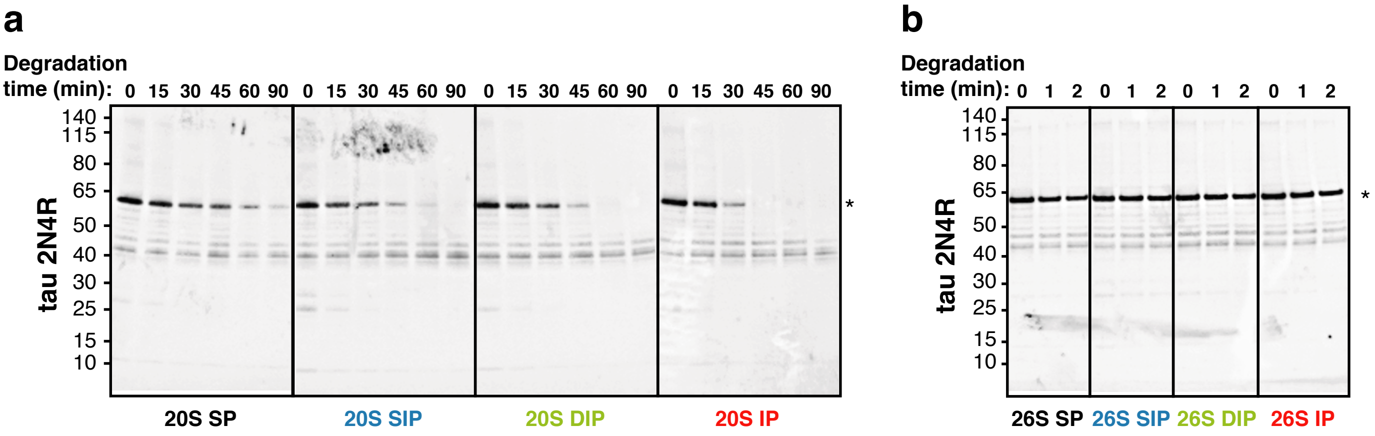

Supplementary Figure 14. Full-length images for the western blots included in Figure 7. (**a**) Western blot analysis of the kinetics of degradation of tau by the four 20S proteasome subtypes. The star indicates the expected band for tau 2N4R isoform. (**b**) Western blot analysis of the kinetics of degradation of tau by the four 26S proteasome subtypes.The star indicates the expected band for tau 2N4R isoform.
